# Context Specific and Differential Gene Co-expression Networks via Bayesian Biclustering

**DOI:** 10.1371/journal.pcbi.1004791

**Published:** 2016-07-28

**Authors:** Chuan Gao, Ian C. McDowell, Shiwen Zhao, Christopher D. Brown, Barbara E. Engelhardt

**Affiliations:** 1 Department of Statistical Science, Duke University, Durham, North Carolina, United States of America; 2 Program in Computational Biology and Bioinformatics, Duke University, Durham, North Carolina, United States of America; 3 Department of Genetics, University of Pennsylvania, Philadelphia, Pennsylvania, United States of America; 4 Department of Computer Science, Center for Statistics and Machine Learning, Princeton University, Princeton, New Jersey, United States of America; University of Southern California, UNITED STATES

## Abstract

Identifying latent structure in high-dimensional genomic data is essential for exploring biological processes. Here, we consider recovering gene co-expression networks from gene expression data, where each network encodes relationships between genes that are co-regulated by shared biological mechanisms. To do this, we develop a Bayesian statistical model for *biclustering* to infer subsets of co-regulated genes that covary in all of the samples or in only a subset of the samples. Our biclustering method, *BicMix*, allows overcomplete representations of the data, computational tractability, and joint modeling of unknown confounders and biological signals. Compared with related biclustering methods, BicMix recovers latent structure with higher precision across diverse simulation scenarios as compared to state-of-the-art biclustering methods. Further, we develop a principled method to recover context specific gene co-expression networks from the estimated sparse biclustering matrices. We apply BicMix to breast cancer gene expression data and to gene expression data from a cardiovascular study cohort, and we recover gene co-expression networks that are differential across ER+ and ER- samples and across male and female samples. We apply BicMix to the Genotype-Tissue Expression (GTEx) pilot data, and we find tissue specific gene networks. We validate these findings by using our tissue specific networks to identify trans-eQTLs specific to one of four primary tissues.

## Author Summary

Recovering gene co-expression networks from high-throughput experiments to measure gene expression levels is essential for understanding the genetic regulation of complex traits. It is often assumed for simplicity that gene co-expression networks are static across different contexts—e.g., drug exposure, genotype, tissue, age, and sex. The biological reality is that, along with differences in gene expression levels, there are differences in gene interactions across contexts. In this work, we describe a model for Bayesian biclustering, or recovering non-disjoint clusters of co-expressed genes in subsets of samples using gene expression level data. Using results from our biclustering model, we build gene co-expression networks jointly across all genes by computing the full regularized covariance matrix between all pairs of genes instead of testing each possible edge separately. Because biclustering recovers structure in subsets of the samples, we are able to recover gene co-expression networks that occur across all samples, that are differential across contexts (e.g., up-regulated in males and down-regulated in females), and that are unique to a context (e.g., only co-expressed in lung tissue). We illustrate the robustness of our approach and biologically validate the networks recovered from three different gene expression data sets.

## Introduction

Cellular mechanisms tightly regulate gene transcription. Gene transcription is not independently regulated across genes: many of the mechanisms regulating transcription affect multiple genes simultaneously. Functional *gene modules* consist of subsets of genes that share similar expression patterns and perform coordinated cellular functions [1, 2]. This cluster-based description of gene expression fails to capture the informative co-expression patterns among genes within a gene module.

If we consider each gene as a vertex in a network, then pairs of genes within a gene module for which the correlation in expression levels cannot be explained by other genes may be connected by an undirected edge. Across all genes, these pairwise relationships constitute gene co-expression networks. Constructing these undirected gene networks, as compared to clustering genes into gene modules [3–6], provides rich detail about pairwise gene relationships. An even richer structure capturing these pairwise relationships would be a directed network of genes, but currently directed networks are computationally intractable to construct relative to undirected gene networks [7–10]. Our work describes a rigorous approach to recover undirected gene co-expression networks from gene expression data that uses a probabilistic latent factor model to quantify the relationships between all pairs of genes.

Several methods have been proposed to construct gene co-expression networks by partitioning a set of genes (and, in some cases, samples) into gene modules from which an undirected graph is elicited [11–14]. In most cases, gene partitioning creates disjoint sets of genes, implying that genes only participate in a single gene module. Biologically this assumption does not hold; the impact is that the gene networks built from methods that assume disjoint clusters are modular. These approaches are not probabilistic, and thus uncertainty in the network edges is not well characterized.

Alternatively, statistical latent factor models are often used to identify groups of co-regulated genes in gene expression data [15–18]. In particular, latent factor models decompose a matrix **Y** ∈ ℜ^*p*×*n*^ of *p* genes and *n* samples into the product of two matrices, **Λ** ∈ ℜ^*p*×*K*^, the factor loadings, and **X** ∈ ℜ^*K*×*n*^, the latent factor matrix with *K* latent factors, assuming independent Gaussian noise. Because it is costly to obtain and assay genome-wide gene expression levels in a single sample, most expression studies include observations of many more genes *p* than samples *n* [19, 20]. This so-called *p* ≫ *n* scenario limits our ability to find latent structure in this expansive, underconstrained space. High dimensional data suggests the use of strong regularization on the latent space to provide sufficient structure for the optimization to reach a robust solution. For example, we may regularize a latent space to discourage all but a few genes from contributing to a latent factor through a sparsity-inducing prior or penalty on the loading vectors [15, 21, 22]. Non-disjoint clusters of genes can be extracted from the resulting fitted sparse loading matrix by recovering all genes with non-zero loadings on the same factor [16]. Sparse latent factor models are more interpretable than their non-sparse counterparts because of this clustering effect.

Besides encouraging sparsity in the factor loading matrix, which results in non-disjoint clusters of genes that co-vary across all samples, one can also induce sparsity in the factor matrix, which results in non-disjoint subsets of samples within which subsets of genes uniquely exhibit co-variation. Statistically, this corresponds to regularizing both factor and loading matrices using priors that encourage zero-valued elements. Biologically, such a model recovers components that identify small numbers of correlated genes, where the correlation among the genes is exclusive to, for example, female samples. This statistical model encodes a general framework known as *biclustering* [23–35]. A biclustering model decomposes a matrix into clusters that each correspond to a subset of samples and a subset of features that exhibit latent structure unique to those subsets.

Gene expression levels have been shown to be sensitive to a number of environmental, biological, and technical covariates including experimental batch, sex, ethnicity, smoking status, or sample tissue heterogeneity [36, 37]. Methods to adjust the observation matrix to control the effects of these covariates without eliminating signals of interest have been proposed. Most attempts have been limited to correcting for confounding effects in a two-stage approach [20, 38, 39] or controlling for confounding effects jointly with association testing [40, 41]. The two-stage approach applied to estimates of co-expression networks has not been successful: often variation in expression levels of groups of co-expressed genes are captured in the estimates of confounding effects and controlled in the first stage, leading to false negatives [42].

In this paper, we develop a Bayesian statistical model for biclustering called *BicMix*. Our motivation behind developing this method was to identify large numbers of subsets of co-regulated genes capturing as many sources of gene transcription variation as possible within arbitrary subsets of the samples. Our biclustering model also includes non-sparse components to represent sources of transcription variation that affect all genes or all samples, which includes many types of confounding effects. We developed a simple but principled statistical method to reconstruct gene co-expression networks based on the regularized covariance matrices estimated using our biclustering model. This method recovers different types of gene co-expression networks, categorized by quantifying the contribution of each sample to the latent components: i) ubiquitous co-expression networks, ii) co-expression networks specific to a sample context, and iii) networks with differentially co-expressed genes across a sample context.

In this paper, we motivate and describe our Bayesian model for biclustering, BicMix. We validate our biclustering model on extensive simulations and compare biclustering with a number of state-of-the-art methods. We then apply our model to gene expression data without correcting for known or unknown confounders. In particular, we apply our biclustering model to gene expression levels measured in heterogeneous breast cancer tissue samples to recover a co-expression network that is differentially expressed across estrogen receptor positive and negative (ER+ and ER-) samples [43, 44]. Next, we apply our biclustering model to gene expression levels measured in lymphoblastoid cell lines (LCLs) from a cohort of patients in a cardiovascular disease study to identify co-expression networks with differential co-expression across males and females, and across smoking status [45]. Finally, we apply BicMix to the Genotype-Tissue Expression (GTEx) pilot data to elucidate tissue specific gene co-expression networks [46]. We validate the recovered networks by identifying tissue specific trans-eQTLs using the recovered tissue specific co-expression networks.

## Results

### Bayesian biclustering using BicMix

Biclustering was first introduced to detect clusters of states and years that showed similar voting patterns among Republicans in national elections [47] and was later referred to as *biclustering* in the context of identifying co-expressed genes in subsets of samples [23]. Biclustering has also been referred to as two mode clustering [48], subspace clustering [49, 50], or co-clustering [51] in various applied contexts. Biclustering was used successfully to explore latent sparse structure in different applied domains [52], including gene expression data [23, 24, 53–56], neuroscience [57], time series data [54], and recommendation systems [58]. Refer to comprehensive biclustering reviews for details [27, 59].

Biclustering approaches fall into four general categories. The first category assumes that each observed gene expression level for one sample is a linear combination of a mean effect, a row (gene) effect, and a column (sample) effect, some of which may be zero [23]. One approach in this category, *Plaid*, captures gene expression levels as a sum of many sparse submatrix components, where each submatrix includes non-zero values only for a subset of genes and subset of samples [30, 31]. The second category of biclustering methods uses hierarchical clustering to group together similar samples and features [3]. For example, samples may be clustered by considering some measure of feature similarity [24, 25, 28, 32, 35]. The third category of biclustering methods builds up biclusters by iteratively grouping features in a greedy way—e.g., identifying all genes that have correlated expression levels with a selected gene—and then removing samples that do not support that grouping [26]. The last category of biclustering methods uses Bayesian sparse factor analysis models [33]. These models decompose a gene expression matrix into two sparse matrices. Sparsity-inducing priors, such as the Laplace prior, are imposed on elements of both the loading and the factor matrices to induce zero-valued elements. *K* biclusters are recovered as the non-zero feature and sample components for each of the *K* latent components. Our approach falls into this last category of a sparse latent factor model for biclustering.

#### BicMix: Bayesian biclustering model

We developed a Bayesian biclustering model, *BicMix*, built on factor analysis with sparsity-inducing priors on both of the low dimensional matrices. In particular, we defined the following latent factor model for matrix **Y** ∈ ℜ^*p*×*n*^, which is the set of observations of *p* gene expression levels across *n* samples:

Y=ΛX+ϵ (1)

where **Λ** ∈ ℜ^*p*×*K*^ is the *loading matrix*, **X** ∈ ℜ^*K*×*n*^ is the *factor matrix*, ***ϵ*** ∈ ℜ^*p*×*n*^ is the residual error matrix, and *K* is fixed a priori. We assume that the residual error is independent across genes and samples and has a zero-mean multivariate Gaussian distribution with gene specific variance: $\epsilon_{\cdot ,i}∼N_{p}(0,\Psi )$ for *i* = 1, …, *n*, where **Ψ** = diag(*ψ*_1_, …, *ψ*_*p*_). While a value must be specified for *K*, the number of latent factors, this model removes factors that are unsupported in the data through a global sparsity-inducing prior, so *K* should be set as an overestimate of the number of latent factors (see Methods) [60].

To induce sparsity in both the factors (samples) and the loadings (genes), we used the three parameter beta (TPB) distribution [61], which has been shown to be computationally efficient and to induce flexible modeling behavior. In previous work [60, 62], we included three layers of regularization via the TPB distribution to induce sparsity in the loading matrix. Here, we extended this model to include this same sparsity-inducing prior on the factor matrix (see Methods). With a flexible sparsity-inducing prior on both the factor and loading matrices, the model becomes a biclustering model, estimating subsets of genes for which co-variation is observed in a subset of samples. This structured prior produces favorable behavior in this biclustering model: i) the number of factors and loadings are effectively estimated from the data, because the sparsity-inducing prior removes unused factors; ii) each factor and corresponding loading has a different level of shrinkage applied to it, enabling a non-uniform level of sparsity and corresponding percentage of variance explained (PVE) for each factor and loading pair [60]; iii) neither the clusters of genes nor the clusters of samples are disjoint, so all genes and all samples may be in any number of clusters, or none; and iv) strong regularization allows overcomplete estimates of the response matrix, with possibly more factors *K* than samples *n* or genes *p*.

**Fig 1:**
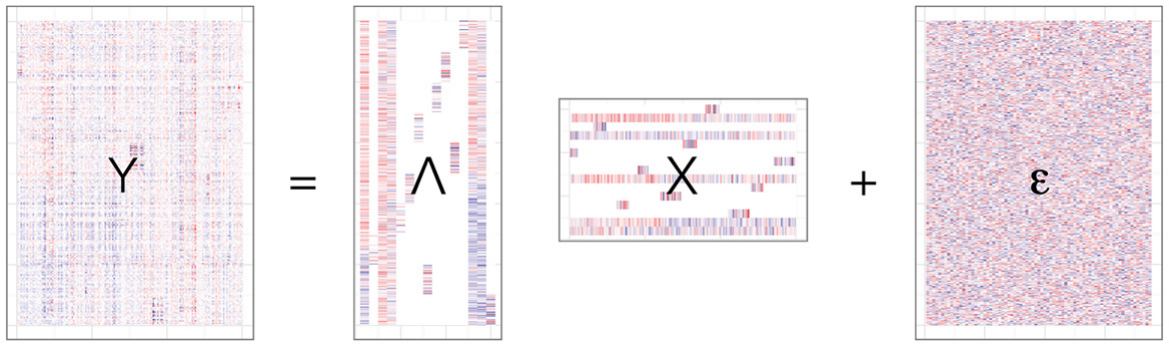
Schematic representation of the BicMix biclustering model. Ordered from left to right are, respectively, the *p* × *n* gene expression matrix **Y**, the *p* × *K* loading matrix Λ including both sparse and dense columns, the *K* × *n* factor matrix **X** including both sparse and dense rows, and the *p* × *n* residual matrix *ϵ*. Blue, red, and white entries in each matrix correspond to negative, positive, and zero values, respectively.

In gene expression data, observed covariates or unobserved confounding effects may systematically influence variation in the observation [38, 40, 41]. As in prior work, we tailored our sparsity-inducing prior to jointly model these often dense confounding effects [60]. In particular, we adapted our model so that the loadings and factors are drawn from a two-component mixture distribution, where each vector is either *sparse*—with zero elements—or *dense*—with no zero elements or all zero elements (Fig 1). We extracted information about whether a vector is sparse or dense directly from the fitted model parameters using the posterior mean of the mixture component assignment variables for each component *k* = 1, …, *K*, where *z*_*k*_ ∈ {0, 1} indicates a dense or a sparse loading and *o*_*k*_ ∈ {0, 1} indicates a dense or a sparse factor. This two-component mixture in the prior distribution for the factors and loadings adds two favorable behaviors to the biclustering model. First, it jointly models covariates that regulate variation in most genes and also in few genes; we have found that confounding effects are often captured in the dense components as all samples and most genes are affected (e.g., batch effects, population structure) [37, 60]. Second, the mixture component has the effect of relaxing a computationally intractable space, enabling scalable parameter estimation in a Bayesian framework. Specifically, considering all possible subsets of genes and samples to identify biclusters is an intractably difficult problem; however, it is computationally tractable to first search over the space for which cluster membership is relaxed, represented as a continuous value between 0 and 1. Then we identify clusters by iteratively shrinking the small magnitude membership values to zero, which maps the continuous values to the binary representation of cluster membership. We estimated parameters in this model using both Markov chain Monte Carlo (MCMC) and a variational expectation-maximization (VEM) approach (see Methods).

#### Gene co-expression networks from biclusters

To construct an undirected gene network, we built a Gaussian Markov random field, or a Gaussian graphical model (GGM) [63], using the components estimated with our biclustering model (see Methods). In particular, regularized estimates of the gene-by-gene covariance matrix *Ω* may be computed from our parameter estimates as **Ω** = **Λ** **Σ** **Λ**^**T**^ + **Ψ**, where **Σ** is the covariance matrix for **X**. Factor analysis is often viewed as a method for low-rank covariance estimation by marginalizing over the factors, **X**. Furthermore, for any subset of components with sparse loading vectors, *A* ⊆ {1, …, *K*}, $\Omega_{A}=\Lambda_{A}\Sigma_{A,A}\Lambda_{A}^{T}+\Psi$, where **Σ**_*A*,*A*_ is the covariance matrix for **X**_*A*_, estimates a regularized covariance matrix for the genes loaded on **Λ**_*A*_. Note that **Ω**_*A*_ is generally both sparse and full rank; biclustering is a highly structured approach to estimating regularized covariance matrices [64]. The inverted covariance matrix is a symmetric precision matrix $\Delta_{A}=\Omega_{A}^{-1}$, where each element *δ*_*j*,*j*′_ can be transformed into the *partial correlation* between genes *j* and *j*′,

$$cor\left(x_{j,\cdot },x_{j^{\prime },\cdot }|x_{\neg (j,j^{\prime }),\cdot }\right)=-\frac{\delta_{j,j^{\prime }}}{\sqrt{\delta_{j,j}\delta_{j^{\prime },j^{\prime }}}},$$

where *x*_¬(*j*, *j*′),⋅_ indicates all genes in *X* that are neither *j* nor *j*′.

In a GGM, edges are defined as pairs of nodes for which the partial correlation is non-zero. Since each loading Λ_*k*_, *k* ∈ *A*, specifies a cluster of genes, we do not invert the full covariance matrix, but instead invert the submatrix that corresponds to genes with non-zero loadings in those components. This approach avoids inducing non-zero precision estimates—and, correspondingly, edges in the GGM—between genes that never occur in the same bicluster. We used GeneNet [63] to test the precision matrix for significant edges. GeneNet assumes that the edges are drawn from a mixture of the null (no edge) and alternative (edge) distributions, *f*(*E*) = *η*_0_ *f*_0_(*E*) + *η*_1_ *f*_1_(*E*), to calculate the probability of each edge being present or not. Practically, we selected edges with a probability > 0.8.

To recover co-variance networks specific to a subset of the samples, where the subset is characterized by context status, we chose the subset of components that contributes to this covariance matrix carefully. In particular, when we select subset *A* to include only components that have non-zero factor values for samples in a specific context (e.g., only female samples), we identify context specific covariance. When we select *A* such that all samples have a non-zero contribution to a component, we recover ubiquitous components. When we select *A* such that the mean rank of the factor values for one sample context is different than the mean rank of factor values for a different sample context—evaluated using a Wilcoxon signed-rank test— we identify components that are differential across the two contexts.

To combine results across runs of biclustering, we extracted co-expression network edges using this procedure separately for each run. Then we used ideas from an ensemble method, bootstrap aggregation (*bagging*) [65], and counted the number of times each edge for a specific network type was recovered across the runs. The final co-expression network contained edges that were identified in ≥ *r* runs.

### Simulation results across biclustering methods

We validated our biclustering model using simulated data sets and compared results from five state-of-the-art biclustering methods. We simulated data from an alternative generative model for observation matrix **Y** = **Λ** **X** + **ϵ**, where **Y** has dimension *p* = 500 by *n* = 300 and $\epsilon_{i,j}∼N(0,\nu^{-1})$. Within this model, we simulated sparsity as follows: for each loading and factor, a number *m* ∈ [5, 20] of elements were randomly selected and assigned values drawn from N(0,2); the remaining elements were set to zero. We allowed components to share as many as five elements. Simulation 1 (Sim1) had ten sparse components. Simulation 2 (Sim2) had ten sparse components and five dense components, for which the loadings and factors were drawn from a N(0,2) distribution. The components were shuffled so that a sparse loading may correspond to a dense factor, and vice versa. For both simulations we considered low and high noise scenarios: the residual variance parameter in the low noise (LN) setting was *ν*^−1^ = 1 and, in the high noise (HN) setting, was *ν*^−1^ = 2. Ten matrices *Y* were generated from each simulation scenario.

We ran BicMix and five other biclustering methods–Fabia [33], Plaid [30], CC [23], Bimax [27], and Spectral biclustering [34]. For all simulations, we ran BicMix by setting *a* = *b* = 0.5, *c* = 1, *d* = 0.5, *e* = *f* = 1 and *ν* = *ξ* = 1 to promote substantial sparsity at the local level (i.e., the horseshoe prior [66]), weaker sparsity at the factor specific level (i.e., the Strawderman-Berger prior [67, 68]), and a uniform prior at the global level of the hierarchy [60]. The algorithm was initialized with warm start values by running MCMC for 500 iterations and using the final sample as the initial state for variational EM. For BicMix results, components that were classified as sparse have each element thresholded at 10^−10^, because our parameter estimation methods converged to values near, but not exactly, zero. All other methods were run using their recommended settings (see Methods). For Sim2, we corrected the simulated data for the dense components by controlling for five principal components (PCs) before all other methods were run; without this initial correction for dense components, results from all five other biclustering methods were uninterpretable. For all runs, BicMix was initialized with *K* = 50 latent factors; all other methods were initialized with the correct number of sparse factors *K* = 10. For Fabia, we ran the software in two different ways. The results from running Fabia with the recommended settings are denoted as *Fabia*. We also set the sparsity threshold in Fabia to the number (from 100 quantiles of the uniform distribution over [0.1, 5]) that produced the closest match in the recovered matrices to the number of non-zero elements in the simulated data; we label these results *Fabia-truth*.

We used the *recovery and relevance score* (R&R score) [27] to measure the false discovery rate (FDR) and sensitivity of each method in recovering true biclusterings. Let the true set of sparse matrices be **M**_1_ and the estimated set of sparse matrices be **M**_2_; then the R&R score is calculated as:

$$\text{Rec}=\frac{1}{|M_{1}|}\sum_{b_{1}\in M_{1}}\max_{b_{2}\in M_{2}}\frac{b_{1}\cap b_{2}}{b_{1}\cup b_{2}},$$ (2)

$$\text{Rel}=\frac{1}{|M_{2}|}\sum_{b_{2}\in M_{2}}\max_{b_{1}\in M_{1}}\frac{b_{1}\cap b_{2}}{b_{1}\cup b_{2}}.$$ (3)

*Recovery* quantifies the proportion of true clusters that are recovered (i.e., recall); *relevance* refers to the proportion of true clusters identified in the recovered clusters (i.e., precision). For BicMix, we applied this R&R score to the components for which both the loading and the factor vectors were sparse, which indicates a bicluster. For the doubly-sparse latent factor models, Fabia and BicMix, we also calculated a sparse stability index (SSI) [60] to compare the recovered and true matrices; SSI is invariant to label switching and scale, and falls between [0, 1] with 1 indicating perfect recovery.

**Fig 2:**
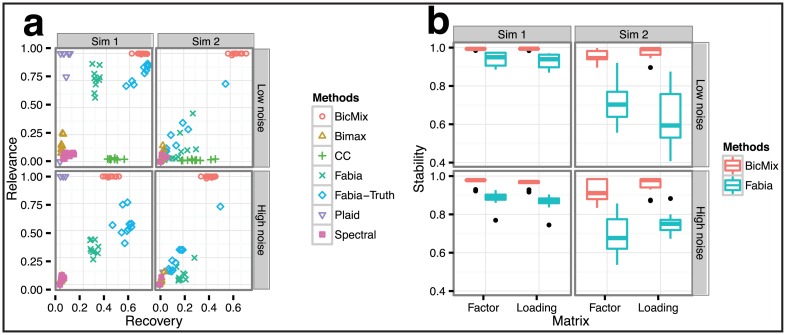
Comparison of BicMix with related methods. Top row: Simulation with low noise. Bottom row: Simulation with high noise. Left column: Sim1 with only sparse components. Right column: Sim2 with sparse and dense components. Panel a: Recovery score on the x-axis, relevance score on the y-axis for all methods in the legend. Panel b: Stability statistic (y-axis) for the sparse components recovered by BicMix and Fabia.

For Sim1, we found that BicMix recovered the sparse loadings, sparse factors, and the biclusters well in the low noise scenario based on both R&R (Fig 2a) and SSI (Fig 2b). Fabia-truth had the second best performance based on R&R. For comparison, Fabia-truth achieved better R&R scores than Fabia (Fig 2a); the clustering results from BicMix dominated those from Fabia-truth, although there was only a small gain in relevance in the low noise Sim1 results for BicMix. Plaid showed high relevance for the recovered biclusters regardless of the noise level for Sim1, but at the expense of poor recovery scores. The remaining methods did not perform well in these simulations with respect to the R&R score for both low and high noise simulation scenarios.

For Sim2, BicMix correctly identified the sparse and dense components (Fig 2a), where a threshold of 〈*z*_*k*_〉 > 0.9 was used to determine when a loading *k* was dense. The performance of Fabia on Sim2 deteriorated substantially relative to its performance on Sim1, although the confounders were removed using principal components (PCs) and the correct number of factors was given. For both BicMix and Fabia, additional noise in the simulation made bicluster recovery more difficult, as shown in deterioration of the recovery score for both methods; however, unlike Fabia, the relevance score of the biclustering from BicMix was unaffected by additional noise in the Sim2 high noise scenario.

The other methods show inferior performance relative to BicMix and Fabia on Sim2. CC assumes that genes in each bicluster have constant expression values, which limits its ability to cluster genes with heterogeneous expression levels. Bimax assumes binary gene expression values (i.e., over- or under-expressed), which limits its utility for heterogeneous expression levels. Spectral biclustering imposes orthogonal constraints on the biclusters; this orthogonality assumption is violated in these simulations and also in gene expression data, where correlated sources of variation may impact similar subsets of genes.

### Application of BicMix to three gene expression study data sets

We now turn to the application of BicMix to gene expression data from three studies. For each application, we first describe the biclustering results from BicMix. Then we show the interpretable networks that were recovered from the BicMix results and discuss network validation using cis- and trans-eQTLs.

#### Breast cancer network

We investigated a breast cancer data set that contains 24,158 genes assayed in 337 breast tumor samples [44, 69, 70] after removing genes that are > 10% missing and imputing missing values of included genes [71] (see Methods). All patients in this data set had stage I or II breast cancer and were younger than 62 years old. Among the 337 patients, 193 had lymph-node negative disease and 144 had lymph-node positive disease; prognostic signatures such as *BRCA1* mutations, estrogen receptor status (ER), distant metastasis free survival (DMFS) were collected for all patients. We focused on building differential gene co-expression networks across ER positive (ER+) and ER negative (ER-) patients because of ER’s prognostic value in profiling breast cancer patients [72]: cancer patients that are ER+ are more likely to respond to endocrine therapies than patients that are ER-. In these data, there are 249 ER+ and 88 ER- patients.

**Fig 3:**
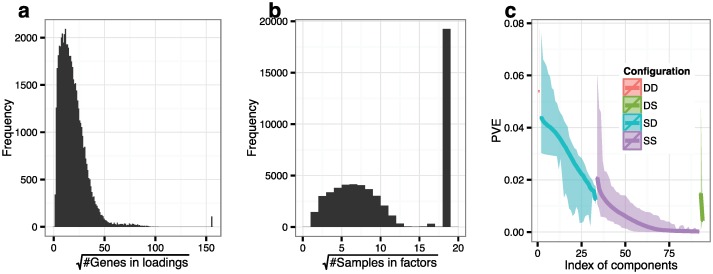
Distribution of the number of genes, the number of samples, and PVE in the breast cancer data. Panel a: Distribution of the number of genes with non-zero values in each of the 53,814 loadings. Panel b: Distribution of the number of samples with non-zero values in each of the 53,814 factors. Panel c: average PVE for the components sorted by PVE within each run. The middle lines show the median PVE, the ribbons show the range of the minimum and maximum PVE across 900 runs. For panels a and b, the peaks on the far right correspond to the number of the genes and samples for the dense loadings and dense factors.

We ran *BicMix* on these data, setting *a* = *b* = 0.5, *c* = 1, *d* = 0.5, *e* = *f* = 0.5 and *ν* = *ξ* = 1 as in the simulations; the initial number of components was set to *K* = 300. Starting from 1,000 random values, BicMix was run until the total number of genes with non-zero loadings across components changed $\leq \frac{Kp}{100}$ over 100 iterations. Removing runs that did not converge, we recovered 53,814 components across 900 runs, of which 110 loadings and 19,239 factors were dense (Fig 3a and 3b).

The distribution of the number of genes in each sparse component was skewed to small numbers (Fig 3a). We categorized each component as one of four configurations: sparse gene loadings with sparse sample factors (SS), sparse gene loadings with dense sample factors (SD), dense gene loadings with sparse sample factors (DS), and dense gene loadings with dense sample factors (DD). SS components captured subsets of genes that are uniquely co-expressed in a subset of samples, which may be due to, e.g., a genotype or environmental context that impacts expression levels of a small number of genes. SD components capture subsets of genes that are differentially co-expressed among all samples, which may be due to, e.g., sex-differential expression or batch effects. DS components capture a subset of samples in which all genes have additional co-variation, which may be due to, e.g., sample contamination. DD components capture variation that occurs across all samples and affects co-variation across all genes, which may be due to, e.g., latent population structure.

For each run, we calculated the percentage of variance explained (PVE) per component *k* as $\frac{\text{Tr}\left(\Lambda_{k}\langle X_{k}X_{k}^{T}\rangle \Lambda_{k}^{T}\right)}{\text{Tr}\left(\Lambda \langle XX^{T}\rangle \Lambda^{T}\right)}$, where *Tr* denotes the trace operator. We ordered the components by PVE within each SS, SD, DS, and DD component category. We calculated the mean, maximum, and minimum values of the PVE-ordered, categorized components across the runs. Note that, because there are no orthogonality constraints, it is possible that many of these components explain similar variation in the observations; for this quantification we are assuming this PVE is disjoint and normalizing across all component-wise PVEs. We also calculated the total PVE explained by each component category by summing the total PVE for all components jointly in each category. The distribution of the number of genes contained in each loading and the PVE by component and across SD, SS, DS, DD categories show that SD and SS components made up the vast majority of recovered components, and that, on average, SD components accounted for a larger PVE than SS components (Fig 3a). The number of components that fell into the SS, SD, DS, DD categories accounted for 64.1%, 35.7%, 0.1%, 0.1%, respectively, of the total number of components. In the same order, components in the four categories accounted for 24.7%, 74.8%, 0.3% and 0.1% of the total PVE.

We selected components from the fitted BicMix model to identify ER+ and ER- specific gene co-expression networks (see Methods). Moreover, to recover gene co-expression networks that are differentially expressed across ER+ and ER- samples, we identified components corresponding to factors that had a significant difference in the mean rank of the factor value between the ER+ and ER- samples based on a Wilcoxon signed-rank test ($p\leq \frac{0.05}{53814}=9.29\times 10^{-7}$). Across our components, we found 996 components unique to ER+ samples, 135 components unique to ER- samples, and 17,051 components differential across ER+ and ER- samples. Interestingly, we note that differential ER status represents the bulk of the SD components recovered across all of the runs. We contrasted the correlation of all of the observed covariates with a representative sample of the SD factors (S1 Fig), and we found that ER status correlated well with many of these SD factors. Tumor grade also correlated well with many of the SD factors; however, tumor grade is somewhat anti-correlated with ER status (S2 Fig), so we chose to focus on ER status because of its clinical utility.

The precision matrices of the subsets of components corresponding to the three network types were constructed and edges among these genes were tested using our method for extracting gene co-expression networks from the fitted biclustering model (see Methods) [63]. For the ER- specific network, we recovered a total of 15 genes and 10 edges that are replicated ≥ 2 times; for the ER+ specific network, we recovered 621 genes and 760 edges that are replicated ≥ 2 times (S3 and S4 Figs; S1 and S2 Tables). We recovered more genes for ER+ than ER- specific networks because there are many more ER+ samples than ER- samples. We found one node and no edges shared across the ER+ and ER- specific networks.

**Fig 4:**
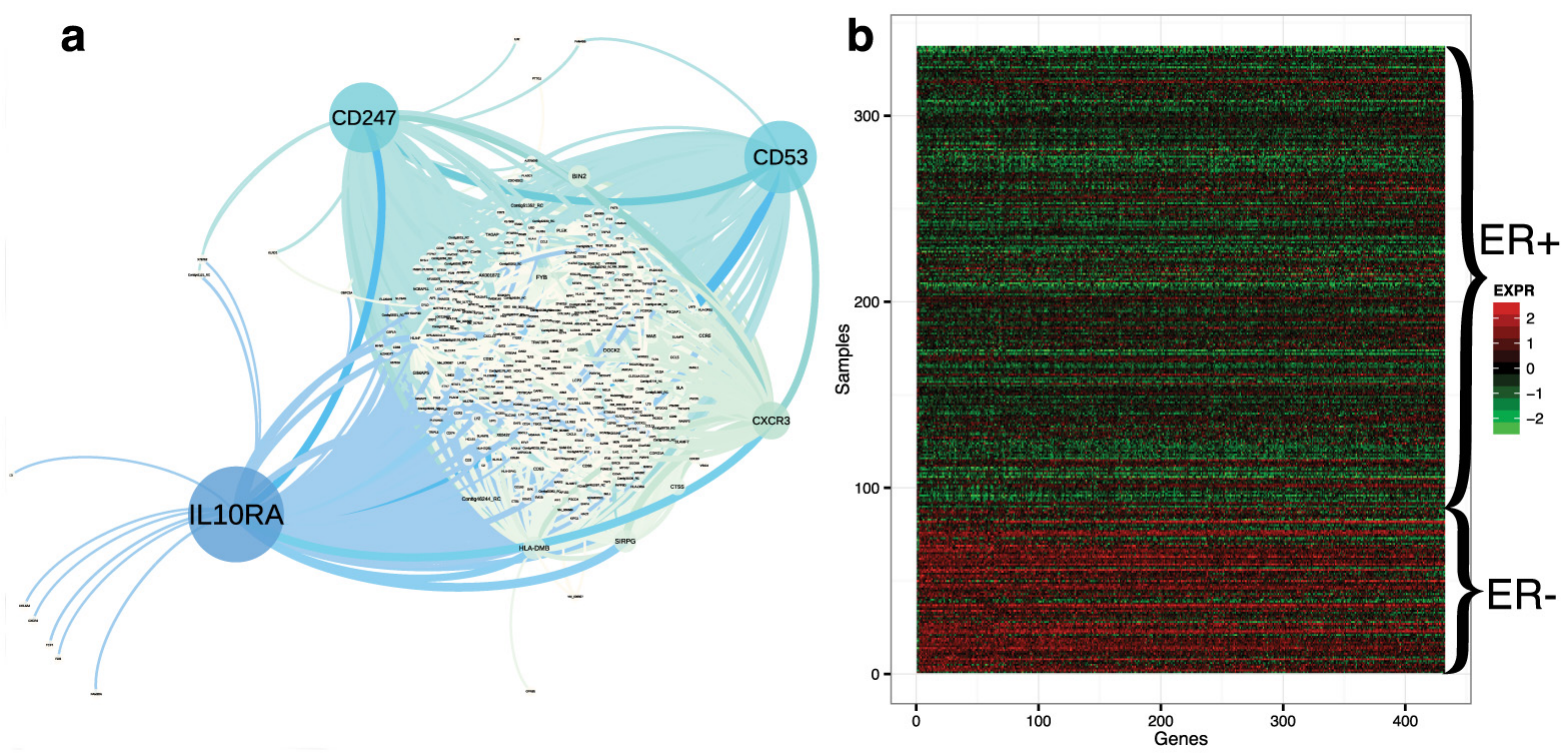
Differential ER-status gene co-expression network and gene expression for ER differential genes. Panel a: differential ER gene co-expression network, where node size corresponds to betweenness centrality, which quantifies the number of shortest paths between all pairs of nodes in the network in which the gene is included. Panel b: gene expression levels for 432 genes in the ER-status differential co-expression network.

For the co-expression network differential across ER+ and ER- samples, we recovered 432 genes and 8,156 edges that were replicated ≥ 15 times across the 900 runs (Fig 4; S3 Table). We hypothesized that, in these differential networks, the 432 genes may be divided into two sub-groups: a group of genes that is up-regulated in the ER+ samples and down-regulated in the ER- samples, and a group of genes that is down-regulated in the ER+ samples and up-regulated in the ER- samples. To explore this hypothesis, we quantified differential expression levels for the 432 genes in the differential gene co-expression network (Fig 4b). We found 430 genes that are up-regulated in ER- samples and down-regulated in ER+ samples. In contrast, we found two genes that are down-regulated in ER- samples and up-regulated in ER+ samples (Fig 4b, first two columns). This bias is likely due to the unbalanced number of ER+ and ER- samples. The genes in the ER-status specific co-expression networks also show a pattern of differential expression (S5 Fig), although not as strong, because gene correlation is differential across networks as opposed to coordinated differential expression levels across a subset of genes.

In the ER-status differential network, we found that many of the annotated genes play critical roles in breast cancer development. For genes that are up-regulated in ER- and down-regulated in ER+ patients:

- *CD53*, the gene that encodes the leukocyte surface antigen *CD53* protein, and *IL10RA*, the gene that encodes the receptor for interleukin 10, belong to a set of three genes (including *DMFS*) that are predictive of outcome specifically in ER tumors [73];
- *CD247* encodes the CD3 zeta chain protein; a previous study has shown that the down-regulation of CD3 zeta expression plays an important role in breast cancer progression [74, 75];
- *CXCR3* encodes the chemokine (*C-X3-C* motif) ligand protein; studies have shown that *CXCR3* deficiency speeds tumor progression [76], and that *CXCR3* isoforms have divergent roles in promoting cancer stem-like cell survival and metastasis [77].

For genes that are up-regulated in ER+ and down-regulated in ER- patients:

- *SFRP2* encodes the secreted frizzled-related protein, which inhibits the growth of triple-negative breast tumors [78];
- *COL12A1* encodes the *alpha chain of type XII collagen*, which has been found to be a regulatory target of *miR-26b* and is predictive of breast cancer recurrence [79].

#### Cardiovascular and Pharmacogenetic (CAP) RNA expression study data

We next applied our biclustering model to a gene expression study with 10,195 expressed genes measured in 480 human lymphoblastoid cell lines (LCLs) [45]. In these data, there are 221 female samples, 259 male samples, 64 smokers, and 416 non-smokers. Age and BMI are also available for each sample. The data were processed according to previous work [45, 60]; In particular, we removed genes with probes on the gene expression array that aligned to multiple regions of the genome using a BLAST analysis against human genome reference hg19, leaving 8,718 expressed genes. No known covariates nor PCs were controlled in these data before projecting each gene expression level to the quantiles of a standard normal distribution (S6 Fig).

We set *K* = 400 and ran EM from 1000 starting points with *a* = *b* = 0.5, *c* = 1, *d* = 0.5, *e* = *f* = 1, *ν* = 1 and *α* = *β* = 1 [60]. After removing runs that did not converge, we recovered a total of 865 runs. On average there were 93 factors across runs, of which on average 21 were dense factors and dense loadings (where both the loading and the factor mixture component posterior probability of being non-sparse ≥ 0.9).

#### Sex-differential networks

**Fig 5:**
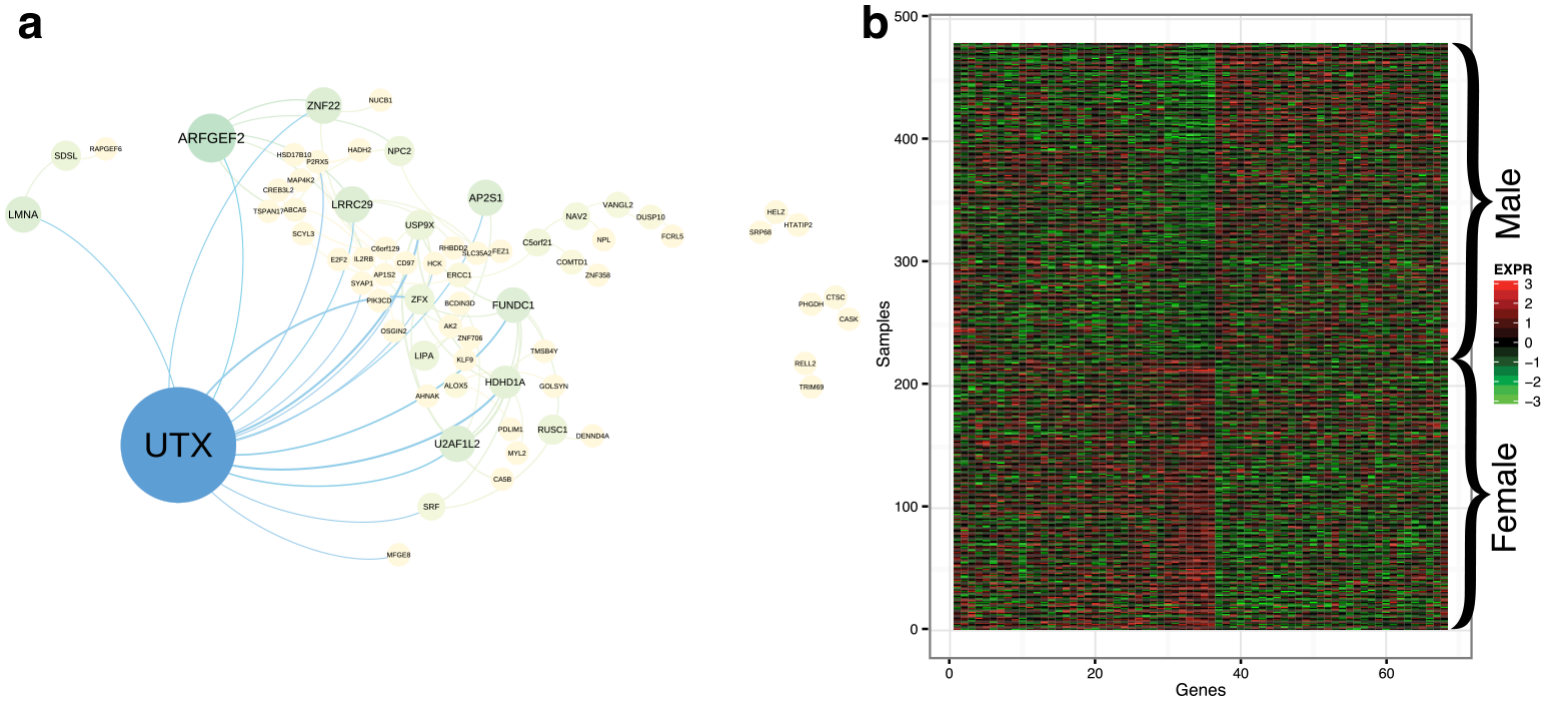
Sex differential gene co-expression network and gene expression levels for sex differential genes. Panel a: differential sex gene co-expression network, where node size corresponds to betweenness centrality. Panel b: gene expression levels for 61 genes in the sex differential gene co-expression network.

To construct a sex differential network, we selected factors that had differential mean values across sex. We recovered 68 genes corresponding to 78 edges that were replicated ≥ 5 times across runs (Fig 5a). The expected number of edges to replicate in this experiment under the null hypothesis is approximately 0.8 (Eq 89). Many of the genes that were identified to have differential co-expression partners across sex play important roles in sex determination or sex specific regulatory activities.

- The hub gene, *UTX*, regulates somatic and germ cell epigenetic reprogramming [80]; more recently it has been shown to be a sex specific tumor suppressor in T-cell acute lymphoblastic leukemia [81];
- *ZFX*, a gene on the human X chromosome that is structurally similar to the Y chromosome gene *ZFY*, has been used for sex identification in many species [82];
- *USP9X*, an X-linked gene, is differentially expressed across sexes in the adult mouse brain, and is hypothesized to play a role in differential neural development in women with XO Turner syndrome [83].

A heatmap of the gene expression levels of these genes across male and female samples shows patterns of differential expression as in the genes highlighted in the breast cancer ER-status differential co-expression network (Fig 5b).

#### Sex specific and smoking status specific networks

**Fig 6:**
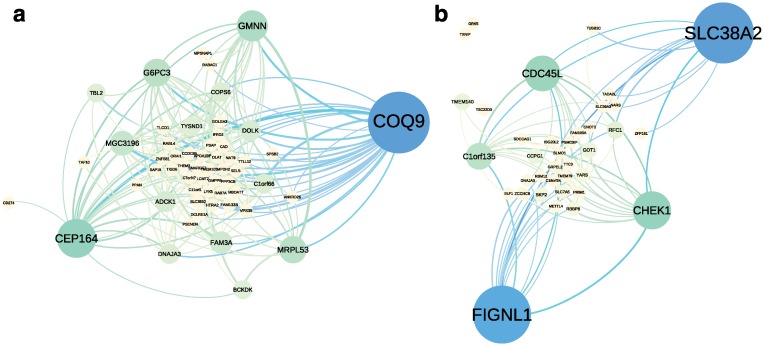
Sex specific gene co-expression networks in the CAP gene expression data. Panel a: Gene co-expression network specific to males. Panel b: Gene co-expression network specific to females. Node size and color correspond to betweenness centrality.

To construct sex specific networks and smoking status specific networks, we identified components with non-zero factor values for either only male or only female samples, or only smokers or only non-smokers, respectively. Using these components, we recovered 57 genes with 176 edges specific to males and 38 genes with 80 edges specific to females, where both networks included edges that were recovered ≥ 10 times across runs (Fig 6). The expected number of edges to replicate in this experiment under the null hypothesis is approximately 2.3 × 10^−7^ (males) and 9.4 × 10^−7^ (females; Eq 89).

For the smoking status specific networks, we identified 31 genes with 36 edges specific to smokers that were recovered ≥ 5 times across runs (S7a Fig); we identified 294 genes with 2,272 edges specific to non-smokers that were recovered ≥ 10 times across runs (S7b Fig). The expected number of edges to replicate in this experiment under the null hypothesis is approximately 4.6 × 10^−7^ (smokers) and 1.3 × 10^−6^ (non-smokers; Eq 89). The decrease in power to detect smoker specific edges compared to non-smoker specific edges is likely due to the imbalanced number of smokers (64) and non-smokers (416) in this study.

#### Genotype-Tissue Expression (GTEx) study

We applied BicMix to a subset of the pilot Genotype-Tissue Expression (GTEx) project data [46]. Our goal for these data was to identify gene co-expression networks that were specific to a single tissue while controlling for networks that appeared across all tissues and other confounding effects. The subset of gene expression data that we used contained *p* = 20,850 genes measured in *n* = 446 samples across four tissues: adipose (*n*_*f*_ = 103), artery (*n*_*a*_ = 118), lung (*n*_*l*_ = 122), and skin (*n*_*s*_ = 106). We preprocessed these RNA-sequencing data as described in Methods. We concatenated these data sample-wise to create a single response matrix **Y** ∈ ℜ^*p* × (*n*_*f*_ + *n*_*a*_ + *n*_*l*_ + *n*_*s*_)^.

We set *K* = 300 and ran EM from 1000 starting points with *a* = *b* = 0.5, *c* = 1, *d* = 0.5, *e* = *f* = 1, *ν* = 1 and *α* = *β* = 1 [60]. After eliminating runs that did not converge, we used a total of 958 runs. In these runs, we recovered on average 119 factors, approximately eight of which were dense in both factors and loadings (S8 Fig).

#### Comparison with biclustering results from comparative methods

To determine whether or not the existing biclustering methods also recover similarly informative biclusters, we ran Fabia, CC, Bimax, Spectral, and Plaid on the GTEx data. To control for confounding effects, we removed the effects of five principal components of the gene expression matrix separately within each tissue so as to maintain the tissue specific effects for all methods except Fabia and BicMix. We found that both Plaid and Fabia were able to separate the tissues in the sample space, as was principal components analysis (PCA; S9 Fig). Starting from 300 biclusters, Plaid reduced the number of biclusters to four, with each bicluster containing samples from a subset of one tissue; adipose samples, however, were not identified uniquely (S10 Fig).

Results from Fabia depended on the specified number of biclusters: when the number of clusters is set to the number of tissues (four), the biclusters distinguished the four tissues (S11 Fig). However, with larger numbers of clusters specified, the separation of the samples by tissue became increasingly obfuscated until, with 20 clusters, there were no tissue specific patterns across clusters (S12 Fig). Compared to results from Plaid and Fabia, the other methods produced uninterpretable results: with 300 clusters, CC found one bicluster that contained all genes and one sample; Bimax failed to recover any biclusters in these data; Spectral found two biclusters, each with zero genes. BicMix, as described above, identified the four tissues in separate clusters as with Fabia (four factors) and PCA (S13 Fig).

#### Tissue specific networks

**Fig 7:**
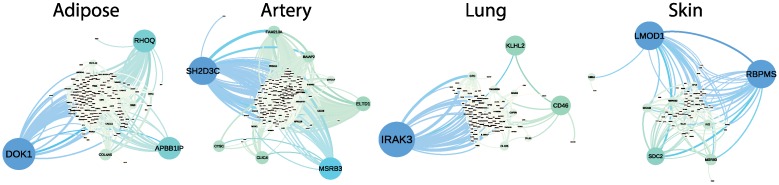
Tissue specific gene co-expression networks in the GTEx pilot data. Adipose: gene co-expression network for adipose. Artery: gene co-expression network for artery. Lung: gene co-expression network for lung. Skin: gene co-expression network for skin. Node size and color correspond to betweenness centrality.

In the BicMix results, we identified all of the bicluster factors and loadings that appeared uniquely in one of the four tissues. We collected genes and edges that were recovered ≥ 10 times across 1000 runs. We recovered 152 genes with 379 edges for adipose tissue, 167 genes with 966 edges for artery tissue, 98 genes with 199 edges for lung tissue, and 70 genes with 171 edges for skin tissue (Fig 7; S4–S7 Tables). The expected number of edges to replicate in this experiment under the null hypothesis is approximately 9.3 × 10^−6^ (adipose), −2.8 × 10^−6^ (artery), 8.2 × 10^−6^ (adipose), 1.7 × 10^−6^ (skin; Eq 89). Note that the negative expectation is due to the approximation in Eq (89).

To study the biological significance of the tissue specific gene clusters, we applied the functional gene annotation tool, DAVID (version 1.1) [84] to perform a gene ontology enrichment analysis on the recovered tissue specific gene clusters from a subset of 100 randomly chosen runs. Using a false discovery rate (FDR) threshold of 0.05, we found 262 (adipose), 265 (artery), 672 (lung), and 560 (skin) *molecular function* Gene Ontology (GO) categories enriched in clusters specific to each of the four tissues (S8–S11 Tables). In particular, the adipose specific gene clusters were most enriched for *lipid biosynthetic process* (FDR ≤ 2.8 × 10^−4^), *lipid metabolism* (FDR ≤ 1.7 × 10^−2^), and *insulin signaling pathway* (FDR ≤ 0.046); Interestingly, artery specific functions also appeared in adipose enriched terms; it has been shown that angiogenesis and vascular functions modulate obesity, adipose metabolism, and insulin sensitivity [85]. The artery specific gene clusters were most enriched for *vascular smooth muscle contraction* (FDR ≤ 9 × 10^−3^), *circulatory system process* (FDR ≤ 0.03), and *ventricular cardiac muscle cell development* (FDR ≤ 0.049). The lung specific gene clusters were enriched for *respiratory burst* (FDR ≤ 5 × 10^−3^) and *Toll-like-receptor* (FDR ≤ 1.8 × 10^−2^), where *Toll-like-receptor* has a role in lung disease [86]. The skin specific gene clusters were most enriched for *melanogenesis* (FDR ≤ 1.9 × 10^−2^), the process of formation of pigmentation, typically of the skin, and *epidermolysis bullosa (EB)* (FDR ≤ 0.03), an inherited connective tissue disease causing blisters in the skin and mucosal membranes. We also observed that many GO terms were enriched in multiple tissues, suggesting that a similar set of biological functions are achieved via different, cell specific mechanisms across tissues.

In the tissue specific networks, we found that the genes with larger betweenness centrality were known to play important roles in tissue specific functions. For adipose tissue, we found that the hub genes play important roles in adipose metabolism and show significant associations with obesity related traits. For example, *DOK1*, the gene that encodes *Docking Protein 1*, was shown to mediate high-fat diet-induced adipocyte hypertrophy and obesity through modulation of *PPAR*-gamma phosphorylation [87]. *APBB1IP*, which encodes the *Rap1-GTP*-interacting adaptor, has been shown to be involved in regulating metabolism and protecting against obesity [88]. *RHOQ*, also known as *TC10*, alters its gene expression levels in visceral adipose tissue of rats that are given a high-fat diet [89]. Genes with smaller betweenness centrality play important adipose specific functions as well; for example, gene expression levels of *PNPLA3* are affected by diet-induced obesity [90], and *LGPAT1* was shown to influence BMI and percent body fat in Native Americans [91].

In the artery specific network, we found that the genes with larger betweenness centrality play important roles in *angiogenesis* and *vasculogenesis*. For example, *ELTD1*, also known as epidermal growth factor (*EGF*), regulates angiogenesis [92]. *CLIC4*, a gene that encodes the chloride intracellular channel protein 4, regulates vasculogenesis through endothelial tube formation; abnormal *CLIC4* expression may play a role in pulmonary arterial hypertension pathology [93]. *CD36* encodes the thrombospondin receptor, and polymorphisms in this gene have significant associations with coronary artery heart disease risk [94]. *BAIAP2* encodes the brain specific angiogenesis inhibitor 1 *BAI1*, and is protective of angiogenesis [95].

In the lung specific network, the genes with larger betweenness centrality play important roles in maintaining the proper function of the lungs or are associated with lung and respiratory diseases. For example, *IRAK3* encodes a member of the interleukin-1 receptor-associated kinase protein family; hospital patients on mechanical ventilation with injury have differential expression of *TLR4* and *IRAK3* [96]. *CD46* encodes a regulatory protein for which higher expression in the lungs of ex-smokers appears to reduce inflammation and protect individuals from emphysema and chronic obstructive pulmonary disease [97, 98]. *IL18RAP* encodes the interleukin 18 receptor accessory protein. A recent study found that genetic variants in *IL1RL1* and *IL18R1* were significantly associated with bronchial hyperresponsiveness [99].

In the skin specific network, we found genes with larger betweenness centrality played critical biological roles in the skin, and were often related to skin tumor growth or carcinoma. For example, *RBPMS* encodes the RNA binding protein with multiple splicing; *RBPMS* is one of six genes that were shared among the top up-regulated genes both in dedifferentiated carcinoma and in carcinoma with loss of 13q [100]. *MCAM* encodes the melanoma cell adhesion molecule; increased expression of *MCAM* in human melanoma cells leads to increased tumor growth and metastasis [101, 102]. Finally, *MSRB* encodes the methionine sulfoxide reductase, which is important for antioxidant repair in human skin [103]. We note that it is possible in the current methodological framework to recover gene networks specific to subsets of the context specific samples; here it is possible that a subset of these skin samples include pre-cancerous cells, which will lead to recovering skin specific networks with edges corresponding to genes that are co-expressed only in pre-cancerous skin tissue.

#### Tissue specific trans-eQTLs

To validate the tissue specific networks identified in the GTEx data, we performed a trans-eQTL analysis on the genes in these networks as follows. An expression QTL (eQTL) is, in this context, a single nucleotide polymorphism (SNP) that regulates expression levels of a specific gene. Transcription regulation may happen in an allele specific way (cis-eQTL) or may be mediated by a non-allele specific process (trans-eQTL). Given a gene *A*, a SNP *Q* that has been identified as a cis-eQTL for gene *A*, and a target gene *B* that is a neighbor of *A* in the tissue specific network, we tested for association between *Q* and *B* in gene expression values derived from samples with matched primary tissue. Using a list of eQTLs identified in previous work [104], we performed a pairwise univariate regression analysis of these eQTLs informed by the tissue specific networks. The same univariate analysis was performed on the permuted gene expression data to find *p* values under the null distribution of no trans-association.

Using our adipose specific networks, we found 40 cis-eQTLs for genes *RHOQ* and *PARVA12*, which were trans-eQTLs for many different target genes in adipose tissue samples (FDR ≤ 0.2). Two of these genes with trans-eQTLs specific to adipose tissue were *TK2* and *EHD2*, which have been associated with the abnormal development of adipose tissues and adipokine levels in mice [105] and lead to specific changes in white adipose tissue of growth hormone receptor-null mice [106], respectively. In artery, we found 121 cis-eQTLs for nine genes that act as trans-eQTLs to multiple target genes. The artery specific trans-eQTL target genes include *CCM2L*, involved in controlling vascular stability and growth [107], and *ARHGEF15*, which affects retinal angiogenesis in endothelial cells [108]. In lung, we found 19 cis-eQTLs for seven genes that are trans-eQTLs for seven target genes including *IRAK3*, described above, and *SIGLEC5*, which has been shown regulate acute pulmonary neutrophil inflammation [109]. In skin samples, we found 13 cis-eQTLs targeting three genes that are trans-eQTLs for two target genes that are unique to skin samples. One of these skin specific trans-eQTLs affects *SLC11A1*, which has been associated with susceptibility to Buruli ulcers [110]. (Full list of tissue specific trans-eQTLs in S12–S15 Tables.)

## Discussion

In this work, we developed a statistical approach to biclustering based on a Bayesian sparse latent factor model. We included a two-component mixture distribution to allow both sparse and dense representations of the features or samples, which captures heterogeneous sources of structured variation within the gene expression data. We used the regularized covariance matrix estimated from the latent factor model to build a Gaussian graphical model with the features represented as nodes in the undirected network. By extracting covariance matrices corresponding to subsets of components, we were able to identify gene co-expression networks that were shared across all samples, unique to a subset of samples, or differential across sample subsets.

We applied our methodology to breast tumor tissue gene expression samples and recovered co-expression networks that are differential across ER+ and ER- tumor types. We applied our methodology to gene expression data from the CAP project and recovered sex-differential, sex specific, and smoking status specific gene co-expression networks. We applied our methodology to the GTEx gene expression pilot data and recovered tissue specific networks for four tissues, which we validated by identifying tissue specific trans-eQTLs.

Factor analysis methods, including the biclustering approach presented here but extending to many other well-studied models, are statistical tools developed for exploratory analyses of the data. In this work, we have exploited the latent structure in both the factor and the loading matrix to estimate the covariance matrix that is specific to sample subsets. We considered tissue type and tumor types, but these methods can be used for any observed binary, categorical, integer, or continuous covariate (e.g., case-control status, batch, sex, age, EBV load). We showed in the Results that the recovered latent structure has substantial context specific biological meaning.

Our results on the GTEx data show that a number of genes were identified as part of multiple tissue specific networks. While individual genes may overlap across networks, the interactions of those genes did not. Genes that co-occurred in multiple tissue specific networks are good candidates to test for differential function across tissues. We also used this approach to study sexual dimorphism, extracting gene networks specific to one sex or differential across the sexes. We showed the potential of this approach to improve statistical power to identify sex specific trans-eQTLs.

In this version of BicMix, extracting a covariance matrix specific to a subset of the samples was performed *post hoc*: the linear projection to the latent space was performed in a mostly unsupervised way, although our three layer sparsity inducing priors add additional structure above SFA-type approaches. As described in the Results, there were multiple categories of gene-interactions that we recovered. These categories included: gene interactions that existed across context, gene interactions that were unique to specific contexts, and gene interactions that were present across contexts but differentially interact in different contexts. However, this approach currently does not use known context to directly inform the projection.

Indirectly, we saw that the sparsity structure on the samples allowed small subsets of the samples to inform projection, but this still relied on a post hoc interpretation of those sample subsets to recover specific network types. Correlation between contexts, such as tissue and batch, or smoking status and sex, would confound these results; here we checked for these correlation among observed covariates (S2 and S6 Figs) and also validated our results using literature searches and trans-eQTL recovery. Furthermore, it may be the case that, for a sample context of interest (e.g., age, sex), there is insufficient signal that is uniquely attributable to those samples (e.g., female) to identify a covariance matrix corresponding to the values of interest in this unsupervised framework. We are currently extending this approach so that the linear projection is explicitly informed by the context of interest.

## Methods

### Bayesian biclustering model for BicMix

We consider the following factor analysis model:

Y=ΛX+ϵ, (4)

where **Y** ∈ ℜ^*p*×*n*^ is the matrix of observed variables, **Λ** ∈ ℜ^*p*×*K*^ is the loading matrix, **X** ∈ ℜ^*K*×*n*^ is the factor matrix, and **ϵ** ∈ ℜ^*p*×*n*^ is the residual error matrix for *p* genes and *n* samples. We assumed $\epsilon_{\cdot ,i}∼N(0,\Psi )$, where **Ψ** = diag(*ψ*_1_, …, *ψ*_*p*_).

In previous work [60, 62], a three parameter beta (TPB) [61] prior was used to model the variance of **Λ**. The three parameter distribution has the form

$$f\left(x:a,b,\phi \right)=\frac{\Gamma (a+b)}{\Gamma (a)\Gamma (b)}\phi^{b}x^{b-1}{\left(1-x\right)}^{a-1}{\left\{1+\left(\phi -1\right)x\right\}}^{-(a+b)},$$ (5)

for *x* ∈ (0, 1), *a* > 0, *b* > 0 and *ϕ* > 0.

We used TPB to induce flexible shrinkage to both **Λ** and **X**. Specifically, we included three layers of shrinkage—global, factor specific, and local—for both the factors and the loadings. Next we describe the sparsity-inducing structure for **Λ** and **X**.

#### Hierarchical structure on Λ

The hierarchical structure for **Λ** is written as

ϱ∼TPB(e,f,ν), (6)

$$\zeta_{k}∼\text{TPB}\left(c,d,\frac{1}{\varrho }-1\right)$$ (7)

$$\varphi_{j,k}∼\text{TPB}\left(a,b,\frac{1}{\zeta_{k}}-1\right),$$ (8)

$$\Lambda_{j,k}∼N\left(0,\frac{1}{\varphi_{j,k}}-1\right).$$ (9)

We used the fact that

$$\varphi ∼\text{TPB}\left(a,b,\nu \right)\Leftrightarrow \frac{\theta }{\nu }∼Be^{\prime }\left(a,b\right)\Leftrightarrow \theta ∼Ga\left(a,\delta \right)\;\text{and}\;\delta ∼Ga\left(b,\nu \right),$$ (10)

where *Be*′(*a*, *b*) and Ga indicate an inverse beta and a gamma distribution, respectively. Making the substitution $\eta =\frac{1}{\varrho }-1,\;\;\phi_{k}=\frac{1}{\zeta_{k}}-1,\;\;\theta_{j,k}=\frac{1}{\varphi_{j,k}}-1$, we get the equivalent hierarchical structure [61]:

γ∼Ga(f,ν), (11)

η∼Ga(e,γ), (12)

$$\tau_{k}∼Ga\left(d,\eta \right),$$ (13)

$$\phi_{k}∼Ga\left(c,\tau_{k}\right),$$ (14)

$$\delta_{j,k}∼Ga\left(b,\phi_{k}\right),$$ (15)

$$\theta_{j,k}∼Ga\left(a,\delta_{j,k}\right),$$ (16)

$$\Lambda_{j,k}∼N\left(0,\theta_{j,k}\right).$$ (17)

We applied a two-component mixture model to jointly model possibly dense confounding effects by letting *θ*_*j*,*k*_ be generated from a mixture of sparse and dense components:

$$\theta_{j,k}∼\pi Ga\left(a,\delta_{j,k}\right)+\left(1-\pi \right)\delta \left(\phi_{k}\right),$$ (18)

where the hidden variable *z*_*k*_, which indicates whether or not loading *k* is sparse (1) or dense (0), is generated from the following beta-Bernoulli distribution:

π|α,β∼Be(α,β) (19)

$$z_{k}|\pi ∼\mathrm{Bern}\left(\pi \right),\;k=\left\{1,\cdots ,K\right\}.$$ (20)

#### Hierarchical structure for X

The hierarchical structure inducing sparsity in **X**, which is structurally identical to that for Λ, is:

$$\varphi ∼Ga(f_{X},\xi ),$$ (21)

$$\chi ∼Ga(e_{X},\varphi ),$$ (22)

$$\kappa_{k}∼Ga\left(d_{X},\chi \right),$$ (23)

$$\omega_{k}∼Ga\left(c_{X},\kappa_{k}\right)$$ (24)

$$\rho_{k,i}∼Ga\left(b_{X},\omega_{k}\right),$$ (25)

$$\sigma_{k,i}∼\pi Ga\left(a_{X},\rho_{k,i}\right)+\left(1-\pi \right)\delta \left(\omega_{k}\right)$$ (26)

$$x_{k,i}∼N\left(0,\sigma_{k,i}\right),$$ (27)

with *σ*_*k*,*i*_ generated from a two component mixture. Here, the hidden variable *o*_*k*_, which indicates whether or not the factor is sparse (1) or dense (0), has the following beta-Bernoulli distribution:

$$\pi_{X}|\alpha_{X},\beta_{X}∼Be\left(\alpha_{X},\beta_{X}\right)$$ (28)

$$o_{k}|\pi_{X}∼\mathrm{Bern}\left(\pi_{X}\right),\;k=\left\{1,\cdots ,K\right\}.$$ (29)

### Variational expectation maximization

Because of the large dimension of matrices to which we apply BicMix, we used approximate methods for parameter estimation. In particular, we used variational expectation maximization (VEM) to estimate values for latent variables and parameters directly from the data in an approximate way. Extending previous work [60], the posterior probability P=p(Λ,X,z,o,Θ|Y) is written as:

$$\begin{array}{cc} P & \propto p\left(Y|\Lambda ,X\right)p\left(\Lambda |z,\Theta_{\Lambda }\right)p\left(X|o,\Theta_{X}\right)p\left(z|\Theta_{\Lambda }\right)p\left(o|\Theta_{X}\right)p\left(\Theta_{\Lambda }\right)p\left(\Theta_{X}\right)\\ & \propto p(Y|\Lambda ,X)P(\Lambda )P(X), \end{array}$$ (30)

where we used **Θ**_Λ_ and **Θ**_*X*_ to denote the set of parameters related to **Λ** and **X**, respectively. Then,

$$\begin{array}{cc} P(\Lambda ) & =p\left(\Lambda |z,\Theta_{\Lambda }\right)p\left(z|\Theta_{\Lambda }\right)p\left(\Theta_{\Lambda }\right)\\ & ={\left[\prod_{j=1}^{p}\prod_{k=1}^{K}N\left(\Lambda_{j,k}|\theta_{j,k}\right)Ga\left(\theta_{j,k}|a,\delta_{j,k}\right)Ga\left(\delta_{j,k}|b,\phi_{k}\right)\right]}^{1_{z_{k}=1}}\\ & \times {\left[\prod_{j=1}^{p}\prod_{k=1}^{K}N\left(\Lambda_{j,k}|\phi_{k}\right)\right]}^{1_{z_{k}=0}}\left[\prod_{k=1}^{K}Bern\left(z_{k}|\pi \right)\right]Beta\left(\pi |\alpha ,\beta \right)\\ & \times \left[\prod_{k=1}^{K}Ga\left(\phi_{k}|c,\tau_{k}\right)Ga\left(\tau_{k}|d,\eta \right)\right]Ga\left(\eta |e,\gamma \right)Ga\left(\gamma |f,\nu \right) \end{array}$$ (31)

and

$$\begin{array}{cc} P(X) & =p\left(X|o,\Theta_{X}\right)p\left(o|\Theta_{X}\right)p\left(\Theta_{X}\right)\\ & ={\left[\prod_{k=1}^{K}\prod_{i=1}^{n}N\left(x_{k,i}|\sigma_{k,i}\right)Ga\left(\sigma_{k,i}|a_{X},\rho_{k,i}\right)Ga\left(\rho_{k,i}|b_{X},\omega_{k}\right)\right]}^{1_{o_{k}=1}}\\ & \times {\left[\prod_{k=1}^{K}\prod_{i=1}^{n}N\left(x_{k,i}|\omega_{k}\right)\right]}^{1_{o_{k}=0}}\left[\prod_{k=1}^{K}Bern\left(o_{k}|\pi_{X}\right)\right]Beta\left(\pi_{X}|\alpha_{X},\beta_{X}\right)\\ & \times \left[\prod_{k=1}^{K}Ga\left(\omega_{k}|c_{X},\kappa_{k}\right)Ga\left(\kappa_{k}|d_{X},\chi \right)\right]Ga\left(\chi |e_{X},\varphi \right)Ga\left(\varphi |f_{X},\xi \right) \end{array}$$ (32)

To derive the variational EM algorithm, we expanded the posterior probability (Eq (30)) and wrote the expected complete log likelihood for parameters related to **Λ**: *Q*(**Θ**_Λ_) = 〈ℓ_*c*_(**Θ**_Λ_,**Λ**|**z**,**X**,**Y**)〉 as:

$$\begin{array}{cc} Q(\Theta_{\Lambda }) & \propto \sum_{j=1}^{p}\sum_{i=1}^{n}\langle \log p(y_{j,i}|\Lambda ,X,\Theta_{\Lambda },z)\rangle \\ & +\sum_{j=1}^{p}\sum_{k=1}^{K}\langle p\left(z_{k}|\Theta_{\Lambda }\right)\log p\left(\Lambda_{j,k}|\Theta_{\Lambda },z_{k}\right)\rangle +\log p\left(\Theta_{\Lambda }\right)\\ & \propto -\frac{p}{2}\ln\left|\Psi \right|-\sum_{j=1}^{p}\sum_{i=1}^{n}\frac{{\left(y_{j,i}-\sum_{k=1}^{K}\Lambda_{j,k}\langle x_{k,i}\rangle \right)}^{2}}{2\psi_{j,j}}+\sum_{j=1}^{p}\sum_{k=1}^{K}\langle 1-z_{k}\rangle \left\{-\frac{1}{2}\ln\phi_{k}-\frac{\Lambda_{j,k}^{2}}{2\phi_{k}}\right\}\\ & +\sum_{j=1}^{p}\sum_{k=1}^{K}\langle z_{k}\rangle \left\{-\frac{1}{2}\ln\theta_{j,k}-\frac{\Lambda_{j,k}^{2}}{2\theta_{j,k}}+a\ln\delta_{j,k}+\left(a-1\right)\ln\theta_{j,k}-\delta_{j,k}\theta_{j,k}\right\}\\ & +\sum_{j=1}^{p}\sum_{k=1}^{K}\langle z_{k}\rangle \left\{b\ln\phi_{k}+\left(b-1\right)\ln\delta_{j,k}-\phi_{k}\delta_{j,k}\right\}+\sum_{k=1}^{K}\left\{\langle z_{k}\rangle \ln\pi +\left(1-\langle z_{k}\rangle \right)\ln\left(1-\pi \right)\right\}\\ & +\sum_{k=1}^{K}\left\{c\ln\tau_{k}+\left(c-1\right)\ln\phi_{k}-\tau_{k}\phi_{k}+d\ln\eta +\left(d-1\right)\ln\tau_{k}-\eta \tau_{k}\right\}\\ & +e\ln\gamma +(e-1)\ln\eta -\gamma \eta +f\ln\nu +(f-1)\ln\gamma -\nu \gamma +\alpha \ln\pi +\beta \ln(1-\pi ), \end{array}$$ (33)

where we used 〈*X*〉 to represent the expected value of *X*.

Similarly, the expected complete log likelihood for parameters related to **X** takes the following form:

$$\begin{array}{cc} Q(\Theta_{X}) & \propto \sum_{j=1}^{p}\sum_{i=1}^{n}\langle \log p(y_{j,i}|\Lambda ,X,\Theta_{X},O)\rangle \\ & +\sum_{k=1}^{K}\sum_{i=1}^{n}\langle p\left(o_{k}|\Theta_{X}\right)\log p\left(x_{k,i}|\Theta_{X},O_{k}\right)\rangle +\log p\left(\Theta_{X}\right)\\ & \propto -\frac{p}{2}\ln\left|\Psi \right|-\sum_{j=1}^{p}\sum_{i=1}^{n}\frac{{\left(y_{j,i}-\sum_{k=1}^{K}\Lambda_{j,k}\langle x_{k,i}\rangle \right)}^{2}}{2\psi_{j,j}}+\sum_{k=1}^{K}\sum_{i=1}^{n}\langle 1-o_{k}\rangle \left\{-\frac{1}{2}\ln\omega_{k}-\frac{\langle x_{k,i}^{2}\rangle }{2\omega_{k}}\right\}\\ & +\sum_{k=1}^{K}\sum_{i=1}^{n}\langle o_{k}\rangle \left\{-\frac{1}{2}\ln\sigma_{k,i}-\frac{\langle x_{k,i}^{2}\rangle }{2\sigma_{k,i}}+a_{X}\ln\rho_{k,i}+\left(a_{X}-1\right)\ln\sigma_{k,i}-\rho_{k,i}\sigma_{k,i}\right\}\\ & +\sum_{k=1}^{K}\sum_{i=1}^{n}\langle o_{k}\rangle \left\{b_{X}\ln\omega_{k}+\left(b_{X}-1\right)\ln\rho_{k,i}-\omega_{k}\rho_{k,i}\right\}+\sum_{k=1}^{K}\left\{\langle o_{k}\rangle \ln\pi_{X}+\left(1-\langle o_{k}\rangle \right)\ln\left(1-\pi_{X}\right)\right\}\\ & +\sum_{k=1}^{K}\left\{c_{X}\ln\kappa_{k}+\left(c_{X}-1\right)\ln\omega_{k}-\kappa_{k}\omega_{k}+d_{X}\ln\chi +\left(d_{X}-1\right)\ln\kappa_{k}-\chi \kappa_{k}\right\}\\ & +e_{X}\ln\varphi +\left(e_{X}-1\right)\ln\chi -\varphi \chi +f_{X}\ln\xi +\left(f_{X}-1\right)\ln\varphi -\xi \varphi +\alpha_{X}\ln\pi_{X}+\beta_{X}\ln\left(1-\pi_{X}\right). \end{array}$$ (34)

To simplify the calculation, we assumed that the joint distribution *p*(*o*_*k*_,*x*_*k*,*i*_) factorizes as *p*(*o*_*k*_)*p*(*x*_*k*,*i*_), implying that corresponding factors’ and loadings’ sparsity statuses are independent.

We computed the MAP estimates for the parameters that encourage sparsity in the Λ matrix, $\hat{\Theta_{\Lambda }}=arg{\text{max}}_{\Theta_{\Lambda }}Q(\Theta_{\Lambda })$. Specifically, we solved equation $\frac{\partial Q(\Theta_{\Lambda })}{\partial \Theta_{\Lambda }}=0$ to find the closed form MAP estimates. The MAP estimate for the *j*th row of **Λ**, Λ_*j*,⋅_, in matrix form, is:

$${\hat{\Lambda }}_{j,\cdot }=Y_{j,\cdot }\psi_{j,j}^{-1}X^{T}{\left(\langle X\psi_{j,j}^{-1}X^{T}\rangle +Z\Theta_{j}^{-1}+\left(I-\langle Z\rangle \right)\Phi^{-1}\right)}^{-1},$$ (35)

where

$$\begin{array}{c} \Theta_{j}=\left(\begin{array}{cccc} \theta_{j,1} & 0 & \cdots & 0\\ 0 & \theta_{j,2} & \cdots & 0\\ \vdots & \vdots & \ddots & \vdots \\ 0 & 0 & \cdots & \theta_{j,K} \end{array}\right),\;\;\Phi =\left(\begin{array}{cccc} \phi_{1} & 0 & \cdots & 0\\ 0 & \phi_{2} & \cdots & 0\\ \vdots & \vdots & \ddots & \vdots \\ 0 & 0 & \cdots & \phi_{K} \end{array}\right),\;\; \end{array}$$ (36)

and

$$\begin{array}{c} Z=\left(\begin{array}{cccc} \langle z_{1}\rangle & 0 & \cdots & 0\\ 0 & \langle z_{2}\rangle & \cdots & 0\\ \vdots & \vdots & \ddots & \vdots \\ 0 & 0 & \cdots & \langle z_{K}\rangle \end{array}\right) \end{array}$$ (37)

and **I** is the identity matrix.

We computed the expected value of **X**, 〈**X**〉, which has the following form:

$$\langle X_{\cdot ,i}\rangle ={\left(\Lambda^{T}\Psi^{-1}\Lambda +\langle O\rangle \Sigma_{i}^{-1}+\left(I-\langle O\rangle \right)\Omega^{-1}\right)}^{-1}\Lambda^{T}\Psi^{-1}Y_{\cdot ,i},$$ (38)

where

$$\begin{array}{c} \Sigma_{i}=\left(\begin{array}{cccc} \sigma_{1,i} & 0 & \cdots & 0\\ 0 & \sigma_{2,i} & \cdots & 0\\ \vdots & \vdots & \ddots & \vdots \\ 0 & 0 & \cdots & \sigma_{K,i} \end{array}\right),\;\;\Omega =\left(\begin{array}{cccc} \omega_{1} & 0 & \cdots & 0\\ 0 & \omega_{2} & \cdots & 0\\ \vdots & \vdots & \ddots & \vdots \\ 0 & 0 & \cdots & \omega_{K} \end{array}\right),\;\; \end{array}$$ (39)

and

$$\begin{array}{c} O=\left(\begin{array}{cccc} \langle o_{1}\rangle & 0 & \cdots & 0\\ 0 & \langle o_{2}\rangle & \cdots & 0\\ \vdots & \vdots & \ddots & \vdots \\ 0 & 0 & \cdots & \langle o_{K}\rangle . \end{array}\right) \end{array}$$ (40)

We computed the expected value of $X\psi_{j,j}^{-1}X^{T}$:

$$\langle X\psi_{j,j}^{-1}X^{T}\rangle =\psi_{j,j}^{-1}\left(\langle X\rangle {\langle X\rangle }^{T}+\Sigma_{X}\right)$$ (41)

where **Σ**_*X*_ denotes the covariance matrix of **X**.

Parameter *θ*_*j*,*k*_ has a generalized inverse Gaussian conditional probability [60, 61], and its MAP estimate is:

$${\hat{\theta }}_{j,k}=\frac{2a-3+\sqrt{{\left(2a-3\right)}^{2}+8\Lambda_{j,k}^{2}\delta_{j,k}}}{4\delta_{j,k}}.$$ (42)

Similarly, the MAP estimate for *σ*_*k*,*i*_ has the following closed form:

$${\hat{\sigma }}_{k,i}=\frac{2a_{X}-3+\sqrt{{\left(2a_{X}-3\right)}^{2}+8\langle x_{k,i}^{2}\rangle \rho_{k,i}}}{4\rho_{k,i}}.$$ (43)

The MAP estimate for *δ*_*j*,*k*_ is:

$${\hat{\delta }}_{j,k}=\frac{a+b-1}{\theta_{j,k}+\phi_{k}}.$$ (44)

Correspondingly,

$${\hat{\rho }}_{k,i}=\frac{a_{X}+b_{X}-1}{\sigma_{k,i}+\omega_{k}}.$$ (45)

Parameter *ϕ*_*k*_ generates both sparse and dense components, and its MAP estimate takes the form:

$${\hat{\phi }}_{k}=\frac{H+\sqrt{H^{2}+MT}}{M},$$ (46)

where

$$H=pb\langle z_{k}\rangle +c-1-\frac{p}{2}\left(1-\langle z_{k}\rangle \right)$$ (47)

$$M=2\left(\langle z_{k}\rangle \sum_{j=1}^{p}\delta_{j,k}+\tau_{k}\right)$$ (48)

$$T=\sum_{j=1}^{p}\Lambda_{j,k}^{2}.$$ (49)

Correspondingly,

$${\hat{\omega }}_{k}=\frac{H_{X}+\sqrt{H_{X}^{2}+M_{X}T_{X}}}{M_{X}},$$ (50)

where

$$H_{X}=nb_{X}\langle o_{k}\rangle +c_{X}-1-\frac{n}{2}\left(1-\langle o_{k}\rangle \right)$$ (51)

$$M_{X}=2\left(\langle o_{k}\rangle \sum_{i=1}^{n}\rho_{k,i}+\kappa_{k}\right)$$ (52)

$$T_{X}=\sum_{i=1}^{n}\langle x_{k,i}^{2}\rangle .$$ (53)

The following parameters have similar updates to *δ*_*j*,*k*_, with simple closed forms because of the conjugacy of the distributions:

$${\hat{\tau }}_{k}=\frac{c+d-1}{\phi_{k}+\eta }$$ (54)

$$\hat{\eta }=\frac{Kd+e-1}{\gamma +\sum_{k}\tau_{k}}$$ (55)

$$\hat{\gamma }=\frac{e+f-1}{\eta +\nu }$$ (56)

The corresponding parameters related to **X** have similar forms:

$${\hat{\kappa }}_{k}=\frac{c_{X}+d_{X}-1}{\omega_{k}+\chi }$$ (57)

$$\hat{\chi }=\frac{Kd_{X}+e_{X}-1}{\varphi +\sum_{k}\kappa_{k}}$$ (58)

$$\hat{\varphi }=\frac{e_{X}+f_{X}-1}{\chi +\xi }.$$ (59)

The expected value of *z*_*k*_|**Θ** is computed as:

$$\begin{array}{cc} \langle z_{k}|\Theta_{\Lambda }\rangle & =p(z_{k}=1|\Theta_{\Lambda })\\ \; & =\frac{\pi \prod_{j=1}^{p}N\left(\Lambda_{j,k}|\theta_{j,k}\right)Ga\left(\theta_{j,k}|a,\delta_{j,k}\right)Ga\left(\delta_{j,k}|b,\phi_{k}\right)}{\left(1-\pi \right)\left(\prod_{j=1}^{p}N\left(\Lambda_{j,k}|\phi_{k}\right)\right)+\pi \prod_{j=1}^{p}N\left(\Lambda_{j,k}|\theta_{j,k}\right)Ga\left(\theta_{j,k}|a,\delta_{j,k}\right)Ga\left(\delta_{j,k}|b,\phi_{k}\right)} \end{array}$$ (60)

The prior on the indicator variable for sparse and dense components, *π*, has a beta distribution, and its geometric mean is the following:

$$\langle \ln\pi \rangle =\psi \left(\sum_{k=1}^{K}\langle z_{k}\rangle +\alpha \right)-\psi \left(K+\alpha +\beta \right)$$ (61)

where *ψ* is the digamma function.

The corresponding parameters related to **X** are

$$\begin{array}{cc} \langle o_{k}|\Theta_{X}\rangle & =p(o_{k}=1|\Theta_{X})\\ & =\frac{\pi \prod_{i=1}^{n}N\left(x_{k,i}|\sigma_{k,i}\right)Ga\left(\sigma_{k,i}|a_{X},\rho_{k,i}\right)Ga\left(\rho_{k,i}|b_{X},\omega_{k}\right)}{\left(1-\pi \right)\left(\prod_{i=1}^{n}N\left(x_{k,i}|\omega_{k}\right)\right)+\pi \prod_{i=1}^{n}N\left(x_{k,i}|\sigma_{k,i}\right)Ga\left(\sigma_{k,i}|a_{X},\rho_{k,i}\right)Ga\left(\rho_{k,i}|b_{X},\omega_{k}\right)}. \end{array}$$ (62)

$$\langle \ln\pi_{X}\rangle =\psi \left(\sum_{k=1}^{K}o_{k}+\alpha_{X}\right)-\psi \left(K+\alpha_{X}+\beta_{X}\right)$$ (63)

Assuming that the residual precision has a conjugate (gamma) prior, $\frac{1}{\psi_{j,j}}∼Ga(1,1)$, then we have

$$\Psi =\text{diag}\left(\frac{{\text{YY}}^{T}-2Y\langle X^{T}\rangle \Lambda^{T}+\Lambda \langle {\text{XX}}^{T}\rangle \Lambda^{T}+2I}{n+2}\right).$$ (64)

In estimating the factors, we invert a *K* × *K* matrix for each sample, and, in estimating the loading matrix, we invert a *K* × *K* matrix for reach gene, so the main source of computational complexity in our algorithm is $O((n+p)K^{3}\;)$. To summarize the description above, we write the complete VEM algorithm for parameter updates:

**Algorithm 1:** Variational expectation maximization for BicMix

**Data:** *Y*, *K*, *n*_*itr*, *a*, *b*, *c*, *d*, *e*, *f*, *a*_*X*_, *b*_*X*_, *c*_*X*_, *d*_*X*_, *e*_*X*_, *f*_*X*_, *α*, *β*,*α*_*X*_, *β*_*X*_

**Initialize starting values:**

Sample *η*, *γ*, *χ*, *φ* ← Ga(1,1)

Sample *π* ← ℬeta(α,β), *π*_*X*_ ← $ℬeta(\alpha_{X},\beta_{X})$,

**for** *j* ← 1 **to** *p* **do**

 Sample *ψ*_*j*, *j*_ ← Ga(1,1)

**for** *k* ← 1 **to** *K* **do**

 Sample *z*_*k*_ ← ℬern(π), *o*_*k*_ ← $ℬern(\pi_{X})$

 Sample *ϕ*_*k*_, *τ*_*k*_, *ω*_*k*_, *κ*_*k*_ ← Ga(1,1)

 **for** *j* ← 1 **to** *p* **do**

  Sample Λ_*j*,*k*_ ← N(0,1), Sample *θ*_*j*,*k*_, *δ*_*j*,*k*_ ← Ga(1,1)

 **for** *i* ← 1 **to** *n* **do**

  Sample *x*_*k*,*i*_ ← N(0,1), Sample *σ*_*ki*_, *ρ*_*k*,*i*_ ← N(0,1)

**for** *t* ← 1 **to** *n*_*itr* **do**

 **for** *j* ← 1 **to** *p* **do**

  Update Λ_*j*, ⋅_ ← Eq (35)

 **for** *k* ← 1 **to** *K* **do**

  Update *θ*_*j*,*k*_ ← Eq (42), *δ*_*j*,*k*_ ← Eq (44)

 **for** *k* ← 1 **to** *K* **do**

  Update *ϕ*_*k*_ ← Eq (46), *τ*_*k*_ ← Eq (54), *z*_*k*_ ← Eq (60)

 Update *η* ← Eq (55), *γ* ← Eq (56), *π* ← Eq (61)

 **for** *i* ← 1 **to** *n* **do**

  Update *X*_⋅,*i*_ ← Eq (38)

  **for** *k* ← 1 **to** *K* **do**

   Update *σ*_*k*,*i*_ ← Eq (43), *ρ*_*k*,*i*_ ← Eq (45)

 **for** *k* ← 1 **to** *K* **do**

  Update *ω*_*k*_ ← Eq (50), *κ*_*k*_ ← Eq (57), *o*_*k*_ ← Eq (62)

 Update *χ* ← Eq (58), *φ* ← Eq (59), *π*_*X*_ ← Eq (63)

 **for** *j* ← 1 **to** *p* **do**

  Update *ψ*_*j*,*j*_ ← Eq (64)

Output **Λ**, **X**, **z**, **o**

Across hundreds of runs, we found that the number of recovered factors was stable within each application with low variance. As a rule of thumb, we initialized the number of latent factors to *K* = 2 × min(*n*, *p*), and, if we find that the number of factors recovered is not reduced by approximately a third, we increased the number of initial factors until we saw this substantial reduction in *K*.

### MCMC: Conditional distributions of BicMix parameters

We derive below the conditional distributions that capture the MCMC approach that we implemented for BicMix. In our manuscript, we used MCMC to compute the warm start parameter settings in the simulations.

We updated the loading matrix **Λ** one row at a time, where each row consists of values across the *K* components. The *j*th row of the loading matrix, Λ_*j*,⋅_, has the following posterior distribution,

$$\Lambda_{j,\cdot }|Y_{j,\cdot }X,\Theta_{i},\psi_{j,j}∼N\left(Y_{j,\cdot }\psi_{j,j}^{-1}X^{T}{\left(X\psi_{j,j}^{-1}X^{T}+V_{j}^{-1}\right)}^{-1},X\psi_{j,j}^{-1}X^{T}+V_{j}^{-1}\right)$$ (65)

where **V**_*j*_ is a *K* × *K* diagonal matrix. If we used *V*_*j*,*k*,*k*_ to denote the (*k*, *k*)th element for **V**_*j*_, then we sampled *V*_*j*,*k*,*k*_ and its related parameters as follows:

$$\begin{array}{c} V_{j,k,k}=\left\{\begin{array}{cc} \theta_{j,k} & \text{if}\;z_{k}=1;\\ \phi_{k} & \text{if}\;z_{k}=0. \end{array}\right. \end{array}$$ (66)

Similarly, each column of **X** consists of values across the *K* components; the *i*th column of the factor matrix, *X*_⋅,*i*_, has the following posterior distribution,

$$X_{\cdot ,i}|Y_{\cdot ,i},\Lambda ,\Sigma_{i},\Psi ∼N\left({\left(\Lambda^{T}\Psi^{-1}\Lambda +W_{i}^{-1}\right)}^{-1}\Lambda^{T}\Psi^{-1}Y_{\cdot ,i},\Lambda^{T}\Psi^{-1}\Lambda +W_{i}^{-1}\right),$$ (67)

where **W**_*i*_ is a *K* × *K* diagonal matrix. If *W*_*i*,*k*,*k*_ denotes the (*k*, *k*)th element of *W*_*i*_, then we sampled the value of *W*_*i*,*k*,*k*_ as follows:

$$\begin{array}{c} W_{i,k,k}=\left\{\begin{array}{cc} \sigma_{k,i} & \text{if}\;o_{k}=1;\\ \omega_{k} & \text{if}\;o_{k}=0. \end{array}\right. \end{array}$$ (68)

We sampled values for the parameters conditional on sparse and dense state as follows. If *z*_*k*_ = 1,

$$\theta_{j,k}|\Lambda_{j,k},\delta_{j,k}∼\text{GIG}\left(a-\frac{1}{2},2\delta_{j,k},\Lambda_{j,k}^{2}\right)$$ (69)

$$\delta_{j,k}|\theta_{j,k},\phi_{k}∼Ga\left(a+b,\theta_{j,k}+\phi_{k}\right)$$ (70)

$$\phi_{k}|\delta_{j,k},\tau_{k}∼Ga\left(pb+c,\sum_{j=1}^{p}\delta_{j,k}+\tau_{k}\right).$$ (71)

If *z*_*k*_ = 0,

$$\phi_{k}|\tau_{k},\Lambda_{j,k}∼\text{GIG}\left(c-\frac{p}{2},2\tau_{k},\sum_{j=1}^{p}\Lambda_{j,k}^{2}\right).$$ (72)

Correspondingly, the following parameters related to **X** were sampled as follows. If *o*_*k*_ = 1

$$\sigma_{k,i}|x_{k,i},\rho_{k,i}∼\text{GIG}\left(a_{X}-\frac{1}{2},2\rho_{k,i},x_{k,i}^{2}\right)$$ (73)

$$\rho_{k,i}|\sigma_{k,i},\omega_{k}∼Ga\left(a_{X}+b_{X},\rho_{k,i}+\omega_{k}\right)$$ (74)

$$\omega_{k}|\rho_{k,i},\omega_{k}∼Ga\left(nb_{X}+c_{X},\sum_{i=1}^{n}\rho_{k,i}+\omega_{k}\right).$$ (75)

If *o*_*k*_ = 0

$$\omega_{k}|\kappa_{k},x_{k,i}∼\text{GIG}\left(c_{X}-\frac{n}{2},2\kappa_{k},\sum_{i=1}^{n}x_{k,i}^{2}\right).$$ (76)

The following parameters are not sparse or dense component specific; they each have a gamma conditional distribution because of conjugacy:

$$\tau_{k}|\phi_{k},\eta ∼Ga\left(c+d,\phi_{k}+\eta \right)$$ (77)

$$\eta |\gamma ,\tau_{k}∼Ga\left(Kd+e,\gamma +\sum_{k=1}^{K}\tau_{k}\right)$$ (78)

γ|η,ν∼Ga(e+f,η+ν). (79)

Parameters related to **X** were sampled as,

$$\kappa_{k}∼Ga\left(c_{X}+d_{X},\omega_{k}+\chi \right)$$ (80)

$$\chi ∼Ga\left(Kd_{X}+e_{X},\varphi +\sum_{k}\kappa_{k}\right)$$ (81)

$$\varphi ∼Ga\left(e_{X}+f_{X},\chi +\xi \right).$$ (82)

The conditional probability for *z*_*k*_ has a Bernoulli distribution:

$$p\left(z_{k}=1|\Theta_{\Lambda }\right)=\frac{\pi \prod_{j=1}^{p}N\left(\Lambda_{j,k}|\theta_{j,k}\right)Ga\left(\theta_{j,k}|a,\delta_{j,k}\right)Ga\left(\delta_{j,k}|b,\phi_{k}\right)}{\left(1-\pi \right)\left(\prod_{j=1}^{p}N\left(\Lambda_{j,k}|\phi_{k}\right)\right)+\pi \prod_{j=1}^{p}N\left(\Lambda_{j,k}|\theta_{j,k}\right)Ga\left(\theta_{j,k}|a,\delta_{j,k}\right)Ga\left(\delta_{j,k}|b,\phi_{k}\right)}.$$

Let *p*_*z*_ = *p*(*z*_*k*_ = 1|**Θ**_Λ_); then the conditional probability for *z*_*k*_ is

$$z_{k}|p_{z}∼Bern\left(p_{z}\right).$$ (83)

The mixing proportion *π* has a beta conditional probability:

$$\pi |\alpha ,\beta ,z_{k}∼Beta\left(\alpha +\sum_{k=1}^{K}1_{z_{k}=1},K-\sum_{k=1}^{K}1_{z_{k}=0}+\beta \right)$$ (84)

where 1 is the indicator function.

Similarly for **X**, the conditional probability for *o*_*k*_ has a Bernoulli distribution:

$$p\left(o_{k}=1|\Theta_{X}\right)=\frac{\pi \prod_{i=1}^{n}N\left(x_{k,i}|\sigma_{k,i}\right)Ga\left(\sigma_{k,i}|a_{X},\rho_{k,i}\right)Ga\left(\rho_{k,i}|b_{X},\omega_{k}\right)}{\left(1-\pi \right)\left(\prod_{i=1}^{n}N\left(x_{k,i}|\omega_{k}\right)\right)+\pi \prod_{i=1}^{n}N\left(x_{k,i}|\sigma_{k,i}\right)Ga\left(\sigma_{k,i}|a_{X},\rho_{k,i}\right)Ga\left(\rho_{k,i}|b_{X},\omega_{k}\right)}.$$ (85)

Let *p*_*X*_ = *p*(*o*_*k*_ = 1|**Θ**_*X*_); then the conditional probability for *o*_*k*_ is

$$o_{k}|p_{X}∼Bern\left(p_{X}\right).$$ (86)

The mixing proportion *π* has a beta conditional probability:

$$\pi_{X}|\alpha_{X},\beta_{X},O_{k}∼Beta\left(\alpha_{X}+\sum_{k=1}^{K}1_{o_{k}=1},K-\sum_{k=1}^{K}1_{o_{k}=0}+\beta_{X}\right)$$ (87)

where 1 is the indicator function.

Finally, we have,

$$\psi_{j,j}∼\text{IG}\left(\frac{n}{2}+1,\frac{\sum_{i=1}^{n}{\left(y_{j,i}-\sum_{k=1}^{K}\Lambda_{j,k}x_{k,i}\right)}^{2}}{2}+1\right).$$ (88)

We implemented the following MCMC algorithm for sampling the parameters of the BicMix model.

**Algorithm 2:** MCMC algorithm for BicMix

**Data:** *Y* *p* × *n* gene expression matrix, *K*, *n*_*itr*

**Initialize parameters**

**for** *t* ← 1 **to** *n*_*itr* **do**

 **for** *j* ← 1 **to** *p* **do**

  **for** *k* ← 1 **to** *K* **do**

   Sample *V*_*j*,*k*,*k*_ according to Eq (66)

  Sample Λ_*j*,⋅_ according to Eq (65)

 **for** *k* ← 1 **to** *K* **do**

  **if** *z*_*k*_ = 1 **then**

   Sample *ϕ*_*k*_ according to Eq (71)

   **for** *j* ← 1 **to** *p* **do**

    Sample *θ*_*j*,*k*_ according to Eq (69), *δ*_*j*,*k*_ according to Eq (70),

  **if** *z*_*k*_ = 0 **then**

   Sample *ϕ*_*k*_ according to Eq (72)

 **for** *k* ← 1 **to** *K* **do**

  Sample *τ*_*k*_ according to Eq (77), *z*_*k*_ with Eq (83)

 Sample *η* according to Eq (78), *γ* with Eq (79), *π* with Eq (84)

 **for** *i* ← 1 **to** *n* **do**

  **for** *k* ← 1 **to** *K* **do**

   Sample *W*_*i*,*k*,*k*_ according to Eq (68)

  Sample *X*_⋅,*i*_ according to Eq (67)

 **for** *k* ← 1 **to** *K* **do**

  **if** *o*_*k*_ = 1 **then**

   Sample *ω*_*k*_ according to Eq (75)

   **for** *i* ← 1 **to** *n* **do**

    Sample *σ*_*k*,*i*_ according to Eq (73), *ρ*_*k*,*i*_ according to Eq (74),

  **if** *o*_*k*_ = 0 **then**

   Sample *ω*_*k*_ according to Eq (76)

 **for** *k* ← 1 **to** *K* **do**

   Sample *κ*_*k*_ according to Eq (80), *o*_*k*_ according to Eq (86)

 Sample *χ* according to Eq (81), *φ* with Eq (82), *π*_*X*_ with Eq (87)

 **for** *j* ← 1 **to** *p* **do**

  Sample *ψ*_*j*,*j*_ according to Eq (88)

Output **Λ**, **X**, **z**, **o**

### Data processing and comparative methods

#### Processing the breast cancer gene expression data

The breast cancer data set is maintained by Dana-Farber Cancer Institute at Harvard University, and is available through their R package: breastCancerNKI version 1.3.1 [70]. We removed genes with > 10% missing values. We imputed the remaining missing values using the R package impute (version 1.36.0) [71, 111]. We projected the gene expression levels of each gene to the quantiles of a standard normal. There were 24,158 genes remaining in the data set after filtering.

#### Processing the CAP gene expression data

The Cardiovascular and Pharmacogenetics (CAP) gene expression data were generated from the Krauss Lab at the Children’s Hospital Oakland Research Institute and is publicly available at Gene Expression Omnimbus (GEO), GSE36868. We used expression levels from 8,718 expressed genes measured in a sample of 480 human immortalized blood cell lines (LCLs) [45]. The data were processed according to previous work [45, 60], including filtering unexpressed genes; however, neither known covariates nor PCs were controlled for before quantile normalization. We also removed genes with probes on the gene expression array that aligned to multiple regions of the genome using a BLAST analysis and human reference genome hg19 in order to remove gene pairs that appeared well correlated due to a co-hybridization effect. We did this because pairs of genes that were artefactually correlated due to co-hybridization were incorrectly identified by BicMix as co-regulated genes, and we wanted to control this artifact.

#### Processing the GTEx gene expression data

We downloaded from dbGaP the Genotype-Tissue Expression (GTEx) project v4 pilot data [46]. The subset of gene expression data that we used contained *p* = 20,134 genes measured in *n* = 446 samples across four tissues: adipose (*n*_*f*_ = 103), artery (*n*_*a*_ = 118), lung (*n*_*l*_ = 122) and skin (*n*_*s*_ = 106). We preprocessed these RNA-seq data as in earlier work and recapitulated here [104].

We trimmed the RNA-seq reads from the GTEx pilot data v4 [46] using Trimmomatic (v.0.30) [112]. We also trimmed the adapter sequences and overrepresented contaminant sequences that are identified by FastQC (v.0.10.1) [113] with 2 seed mismatches and a simple clip threshold of 20. For all reads, we trimmed the leading and trailing nucleotides (low quality or Ns) until a canonical base was encountered with quality greater than 3. For adaptive quality trimming, we scanned the reads with a 4-base sliding window and trimmed when the average quality per base dropped below 20. We discarded sequences shorter than 30 nucleotides. We were left with 3.65 B read pairs in adipose, 4.02 B read pairs in artery, 4.63 B read pairs in lung, 3.63 B read pairs in skin after trimming.

Before mapping the RNA-seq reads to human reference genome assembly GRCh37.p13, we prepared the genome with STAR aligner genomeGenerate mode by setting the splice junction database (sjdbGTFfile) to GENCODE v.19, and setting the splice junction database overhang (sjdbOverhang) to 75 bp. We used default settings for the rest of the parameters. We ran STAR aligner alignReads using default settings except setting outFilterMultimapNmax to 1. We mapped 14.52 B read pairs (91%) uniquely, and we discarded the 1.2 B read pairs (7.5%) that mapped to multiple loci.

We converted the mapped reads to read counts (one read pair per read count) using the software featureCounts [114] on settings that discounted multi-overlapping and chimeric reads. We also required both ends of a read pair to be mapped to the same gene. After filtering all genes with > 25% non-zero values across all tissues, we were left with 20,134 genes. We normalized the read abundance by gene length, GC-content, and library size using the Bioconductor R package cqn [115], and the resulting gene expression values were mapped to the quantiles of the standard normal distribution. Note that we did not correct for confounding factors nor known covariates before quantile normalization. We concatenated these gene expression data sample-wise to create a single response matrix **Y** ∈ ℜ^*p*×(*n*_*f*_ + *n*_*a*_ + *n*_*l*_ + *n*_*s*_)^.

### Simulation comparison

Using these simulated data, we compared BicMix to five other methods: Fabia, Plaid, CC, Bimax, and Spectral biclustering. We ran these methods using the following settings.

For Sim1, we set the number of components to the correct values, and ran each method as follows.

- We ran Fabia (version 2.10.2) using its default parameter settings.
- We ran Fabia-truth using default parameter settings. We set the sparsity threshold in Fabia to the number (from 100 quantiles of the uniform distribution over [0.1, 5]) that produced the closest match in the recovered matrices to the number of non-zero elements in the simulated data.
- We ran Plaid (implemented in the R package biclust [116] version 1.0.2) using background = TRUE to capture the noise, maximum layers were set to 10, number of iterations to find starting values was set to 10, and the number of iterations to find the layers was set to 100.
- We ran CC (implemented in the R package biclust [116] version 1.0.2) by setting the maximum accepted score delta = 1.5 and the scaling factor alpha = 1.0.
- We ran Bimax (implemented in the R package biclust [116] version 1.0.2) by setting the minimum number of rows and columns to 2.
- We ran Spectral biclustering (implemented in the R package biclust [116] version 1.0.2) by setting the normalization method to bistochastization, the number of eigenvalues for constructing the biclusters was set to 10, and the minimum number of rows and columns for the biclusters were set to 2.

For Sim2, we corrected the simulated data for the dense components by controlling for five PCs in the simulated gene expression data, and we ran the methods on the residual matrix as in Sim1, setting the number of components to 10.

### Redundancy of components

We calculated a simple statistic to check the redundancy of the multiple components recovered across multiple runs as follows. For every component in all runs, we counted the number of genes with non-zero values, denoted as *n*_*g*_, and the number of samples with non-zero values, denoted as *n*_*s*_ for each component. We then grouped the components that share the same *n*_*g*_ and *n*_*s*_. For each pair of components in the same group, we counted how many components have non-zero values for the same genes and the same samples (i.e., the *ℓ*_0_ norm between components, or the Manhattan distance). Redundant components corresponded to pairs of factors and loadings for which the Manhattan distance is zero.

### Algorithm for identifying gene co-expression networks from BicMix

We write out the algorithm we used to build the gene co-expression networks using the fitted BicMix model. Note that the sparsity-inducing prior on the covariance matrix of the factors increases the difficulty of computing the gene-wise covariance matrix relative to the common identity matrix covariance in the prior of the factors; however, all of the elements necessary to compute an estimate of the factor covariance matrix have been explicitly quantified in the VEM algorithm already.

**Algorithm 3:** Algorithm to construct gene co-expression network

**Data:** *p* × *K* loading matrix and *K* × *n* factor matrix; Ψ; *net*_*type*, *rep*, *c*, (*d*).

**for** *i* ← 1 **to** *n*_*runs* **do**

 **for** *k*_1_ ← 1 **to** *K* − 1 **do**

  **for** *k*_2_ ← *K*_1_ + 1 **to** *K* **do**

   **if** *cor*(Λ_*k*_1__, Λ_*k*_2__) × *cor*(**X**_*k*_1__, *X*_*k*_2__)> 0.5 **then**

    discard run

 **if** *net*_*type* = *subset specific* **then**

  Add component to *A* when non-zero factors are only in context *c*

 **if** *net*_*type* = *subset differential* **then**

  Compute Wilcoxon signed-rank test for non-zero factor values across contexts *c*, *d* Add component to *A* when *p* < 1 × 10^−10^

 Construct the covariance matrix for **X** as **Σ** ← **〈** **X** **X**^**T**^ **〉** − **〈** **X** **〉** **〈** **X** **〉**^**T**^ (Eqs 41 and 38)

 Calculate the variance for the residual as **Ψ**← Eq (64)

 Construct the precision matrix for subset *A* as $\Delta^{i}={\left(\Lambda_{A}^{i}\Sigma_{A,A}^{i}{\Lambda_{A}^{i}}^{T}+\Psi \right)}^{-1}$

 Run GeneNet [63] on **Δ**^*i*^ to test significance of edges

 Store edges with probability of presence ≥ 0.8

Count number of times each edge is found across all runs

Keep edges that are found ≥ *rep* times

Output the nodes and edges

Draw graph using Gephi [117]

### Quantifying the expected number of edges using ensemble method

We computed the (approximate) expected number of edges to appear *r* or more times at random using our ensemble method as follows. Let *R* represent the total number of runs, *r* represent the threshold for number of runs an edge must appear, *E* represent the total number of possible edges, and $\hat{e}$ represent the average number of edges per run. Note that this is an approximate expectation because we are using $\hat{e}$ instead of the true number of edges recovered in each run; this expectation has a noticeable impact on the result when the variance in edges per run is large (this was the case only in the breast cancer data set), but otherwise lead to a reasonable approximation.

We computed the probability of a single edge occurring in at least *r* networks as follows:

$$\begin{array}{ccc} Pr(|e_{i}|\geq r) & = & 1-\sum_{j=1}^{r-1}Pr(|e_{i}\left|=j\right)\\ Pr(|e_{i}|=j) & = & \left(\begin{array}{c} R\\ j \end{array}\right){\left(\frac{\hat{e}}{E}\right)}^{j}{\left(1-\frac{\hat{e}}{E}\right)}^{R-j}. \end{array}$$

Then the expected number of edges that will occur *r* or more times at random was approximated as follows, assuming edge independence:

$$E_{r}\left[|e|\right]=E\cdot Pr(|e_{i}\left|\geq r\right).$$ (89)

## Supporting Information

S1 Fig Correlation between the sparse loadings (genes), dense factors (samples), and experimental covariates in the breast cancer data. The x-axis represents 30 recovered factors; the y-axis represents the observed covariates; darker blue and red represent large magnitude correlations, whereas white represents no correlation.
(PDF)

S2 Fig Correlation among the known covariates in breast cancer data. The x- and y-axes represents the observed covariates; darker blue and red represent large magnitude correlations, whereas white represents no correlation.
(PDF)

S3 Fig Gene co-expression network specific to ER- samples. Node size corresponds to betweenness centrality.
(PDF)

S4 Fig Gene co-expression network specific to ER+ samples. Node size corresponds to betweenness centrality.
(PDF)

S5 Fig Heatmap of genes identified in the ER+ specific and ER- specific networks. There is evidence of differential expression levels across the two sample types for these genes that are in the ER+ and ER- specific networks.
(PDF)

S6 Fig Correlation among the known covariates in the CAP data. The x- and y-axes represents the observed covariates; darker blue and red represent large magnitude correlations, whereas white represents no correlation.
(PDF)

S7 Fig Smoking status specific gene co-expression networks in the CAP data. Panel a: Gene co-expression network specific to smokers. Panel b: Gene co-expression network specific to non-smokers.
(PDF)

S8 Fig Distribution of the number of genes in loadings and the number of samples in the factors for the GTEx data. Both the sparse and dense loadings and factors are shown. Left: number of genes for all loadings; Middle: number of samples for all factors; Right: a summary of the PVE explained by the components across all runs, where upper bound and the lower bound of the ribbon correspond to the maximum and minimum PVE, and the solid line correspond to the median. The components are inversely sorted by the median.
(PDF)

S9 Fig Principal components analysis applied to the GTEx pilot data. PC1 effectively separates the four tissue types.
(PDF)

S10 Fig Plaid applied to the GTEx pilot data. Each sample point is plotted with jitter to denote the density of each tissue in each included or excluded component. The four factors each capture variation in one (or a subset of one) of the tissues reasonably well, except for adipose.
(PDF)

S11 Fig Fabia with four components applied to the GTEx pilot data. Across factors 1, 2, and 3, the four tissue types are effectively separated.
(PDF)

S12 Fig Fabia with twenty components applied to the GTEx pilot data. With 20 factors, Fabia is no longer able to separate the four tissue types because of limited sparsity.
(PDF)

S13 Fig BicMix applied to the GTEx pilot data. The substantial sparsity induced in BicMix is illustrated in these panels. Note that BicMix separates all four tissues in the first four factors.
(PDF)

S1 Table Genes that are specific to ER- patients. The importance of the genes are ordered by their betweenness centralities.
(TEX)

S2 Table Genes that are specific to ER+ patients. The importance of the genes are ordered by their betweenness centralities.
(TEX)

S3 Table Genes that are differential between ER+ and ER- patients. The importance of the genes are ordered by their betweenness centralities.
(TEX)

S4 Table Genes that are in the adipose-specific network. The genes are ordered by their betweenness centralities.
(TEX)

S5 Table Genes that are in the artery-specific network. The genes are ordered by their betweenness centralities.
(TEX)

S6 Table Genes that are in the lung-specific network. The genes are ordered by their betweenness centralities.
(TEX)

S7 Table Genes that are in the skin-specific network. The genes are ordered by their betweenness centralities.
(TEX)

S8 Table Gene Ontology enrichment analysis across recovered factors for adipose tissue. All term enrichments are for a cutoff of FDR ≤ 0.05. Gene names are suppressed, but the number of genes in each factor is shown.
(TEX)

S9 Table Gene Ontology enrichment analysis across recovered factors for artery tissue. All term enrichments are for a cutoff of FDR ≤ 0.05. Gene names are suppressed, but the number of genes in each factor is shown.
(TEX)

S10 Table Gene Ontology enrichment analysis across recovered factors for lung tissue. All term enrichments are for a cutoff of FDR ≤ 0.05. Gene names are suppressed, but the number of genes in each factor is shown.
(TEX)

S11 Table Gene Ontology enrichment analysis across recovered factors for skin tissue. All term enrichments are for a cutoff of FDR ≤ 0.05. Gene names are suppressed, but the number of genes in each factor is shown.
(TEX)

S12 Table eQTLs for adipose tissue. Associations are listed in the order of the significance of p values.
(TEX)

S13 Table eQTLs for artery tissue. Associations are listed in the order of the significance of p values.
(TEX)

S14 Table eQTLs for lung tissue. Associations are listed in the order of the significance of p values.
(TEX)

S15 Table eQTLs for skin tissue. Associations are listed in the order of the significance of p values.
(TEX)
