# Supplementary figures and images for: Context Specific and Differential Gene Co-expression Networks via Bayesian Biclustering
Source: PLoS Comput Biol. 2016 Jul 28;12(7):e1004791. doi: 10.1371/journal.pcbi.1004791 (PMC4965098; doi:10.1371/journal.pcbi.1004791)

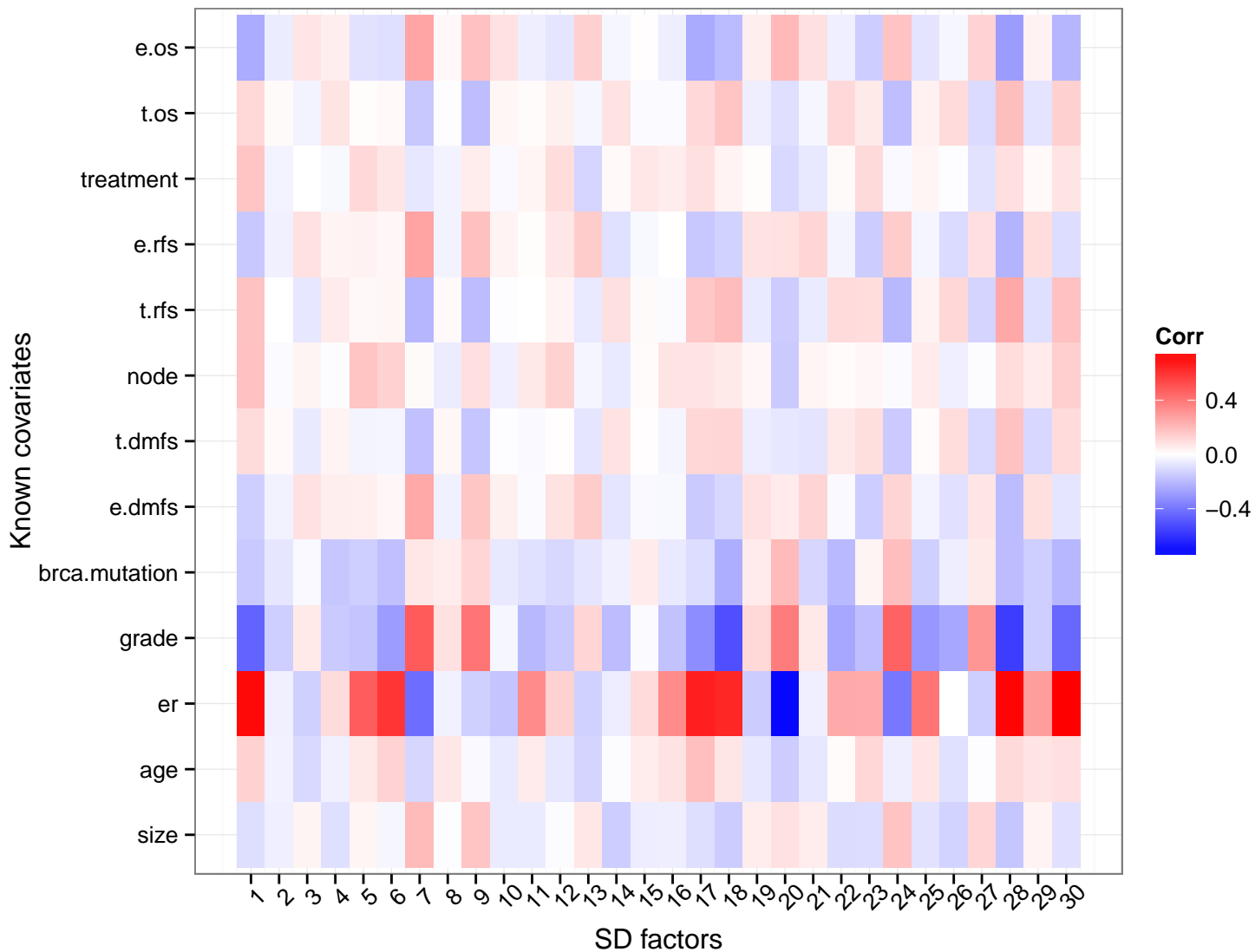

Supplement: S1 Fig — The x-axis represents 30 recovered factors; the y-axis represents the observed covariates; darker blue and red represent large magnitude correlations, whereas white represents no correlation. (PDF) [file pcbi.1004791.s001.pdf]

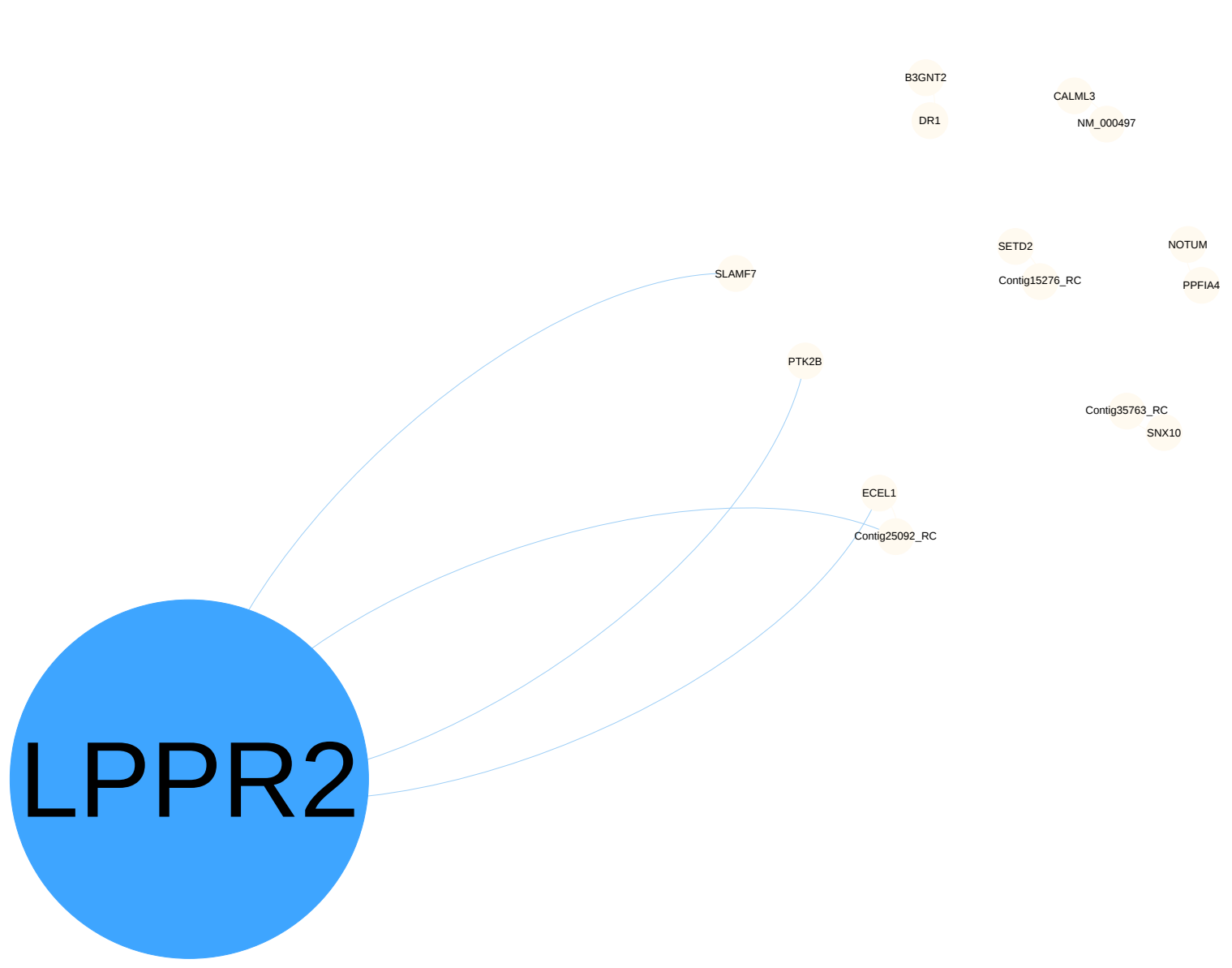

Supplement: S3 Fig — Node size corresponds to betweenness centrality. (PDF) [file pcbi.1004791.s003.pdf]

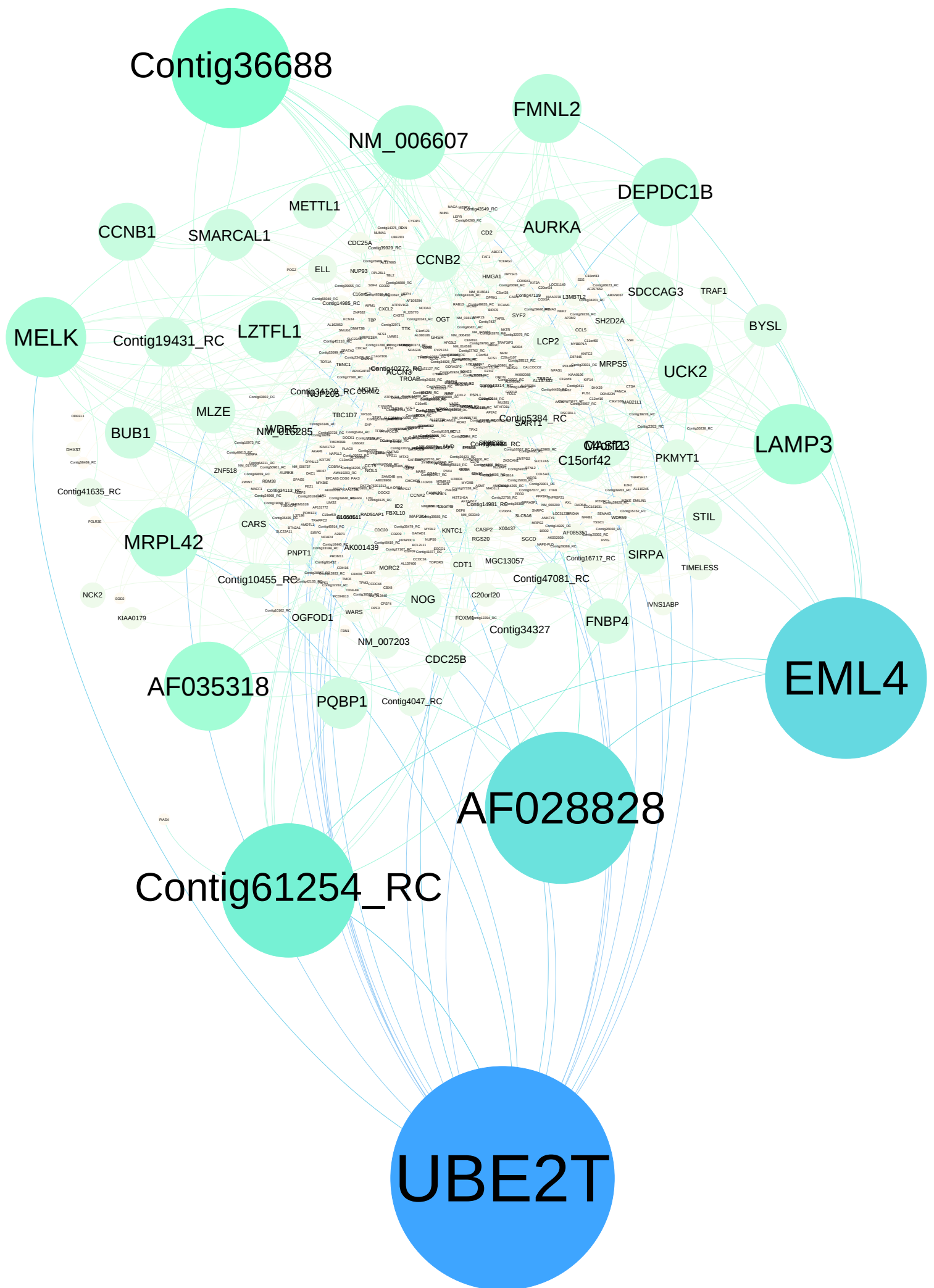

Supplement: S4 Fig — Node size corresponds to betweenness centrality. (PDF) [file pcbi.1004791.s004.pdf]

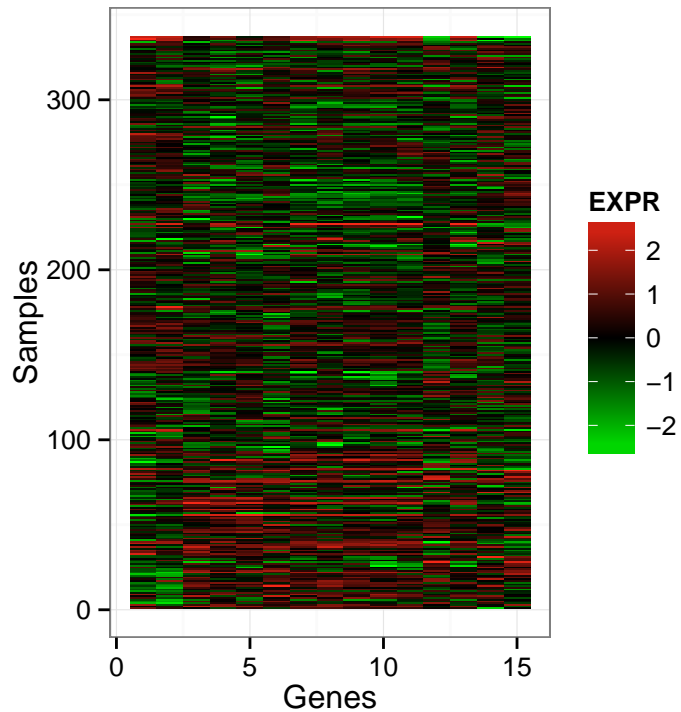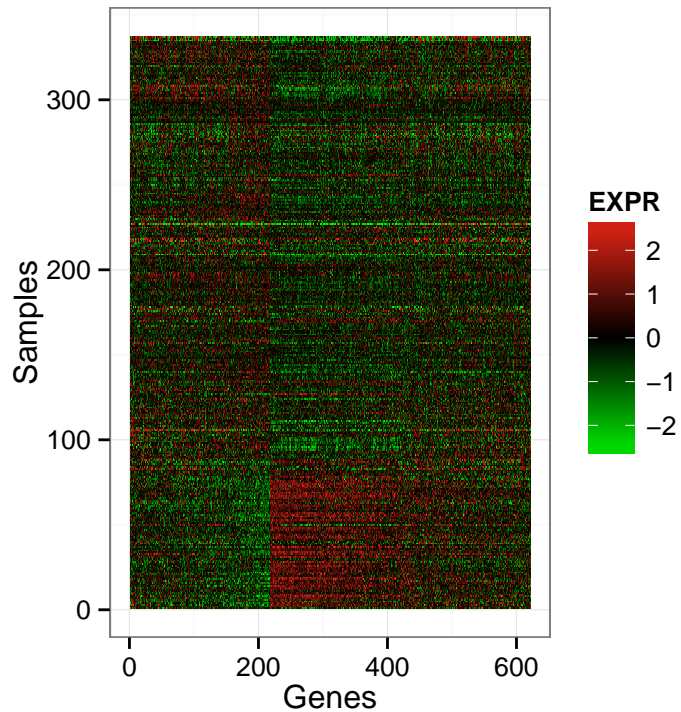

Supplement: S5 Fig — There is evidence of differential expression levels across the two sample types for these genes that are in the ER+ and ER- specific networks. (PDF) [file pcbi.1004791.s005.pdf]

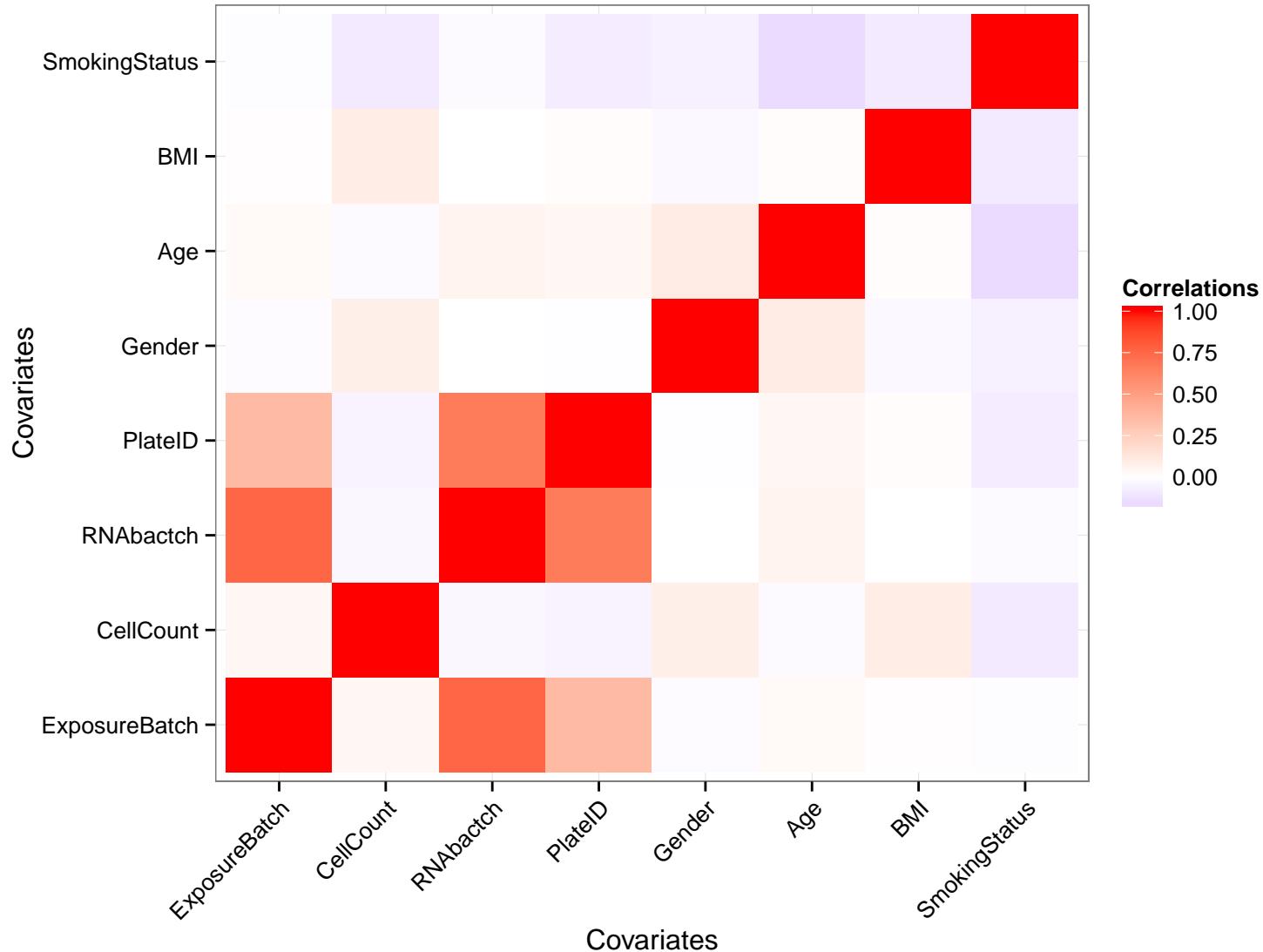

Supplement: S6 Fig — The x- and y-axes represents the observed covariates; darker blue and red represent large magnitude correlations, whereas white represents no correlation. (PDF) [file pcbi.1004791.s006.pdf]

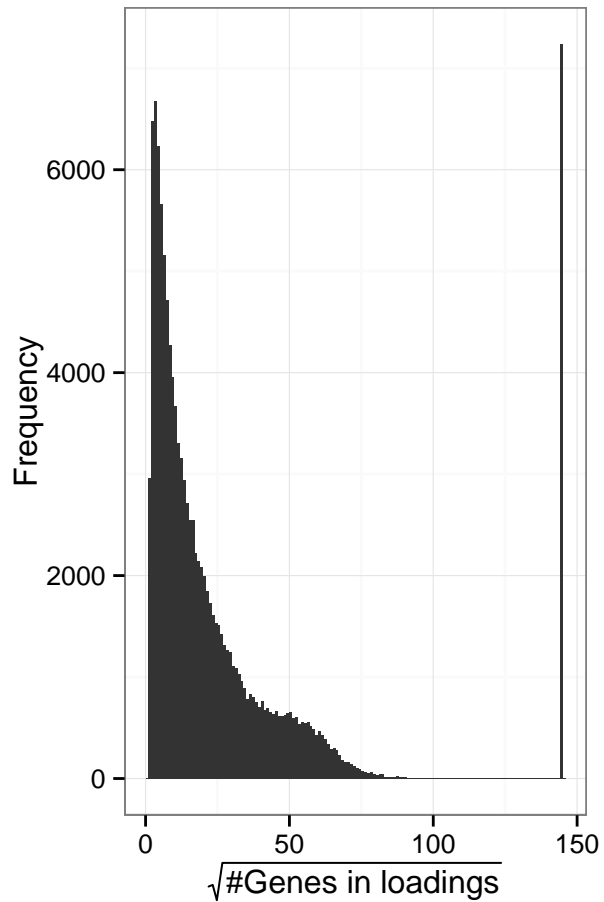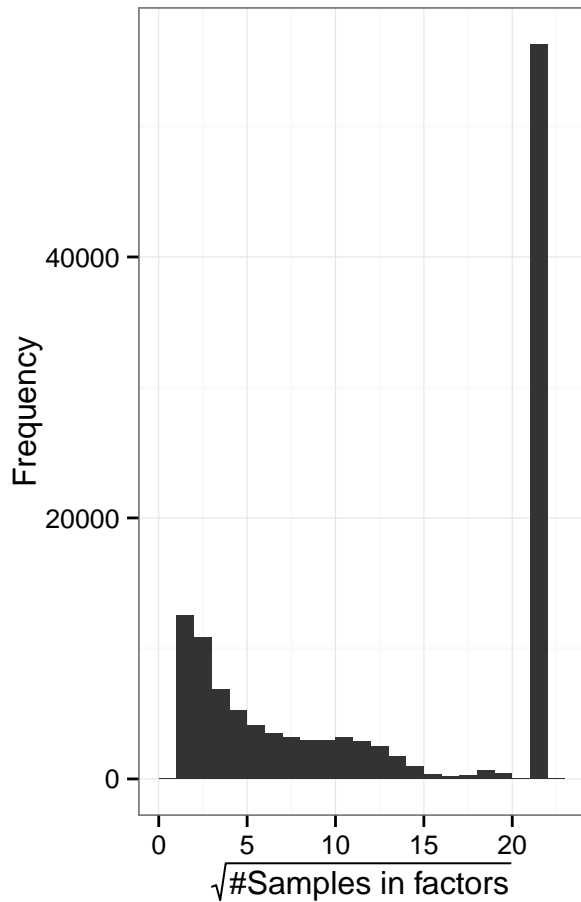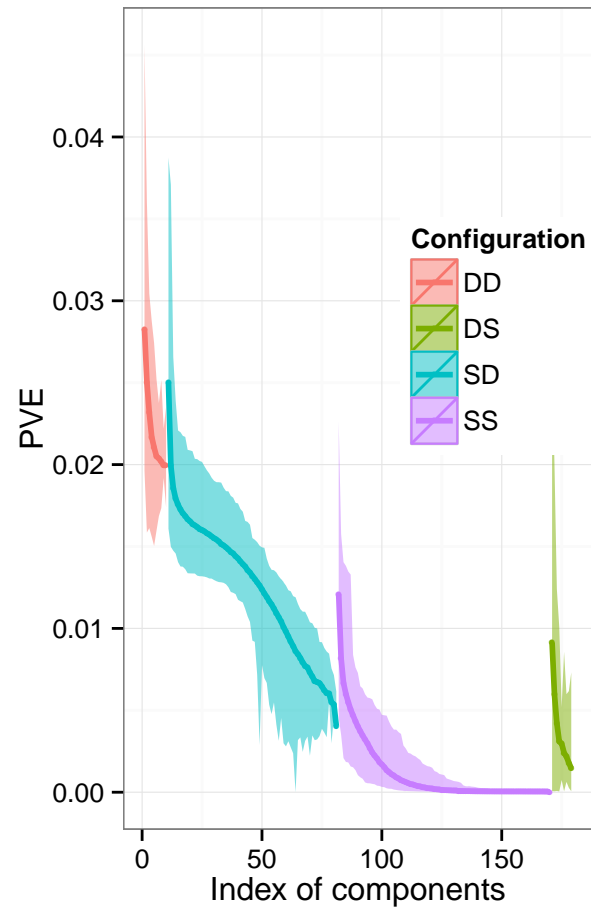

Supplement: S8 Fig — Both the sparse and dense loadings and factors are shown. Left: number of genes for all loadings; Middle: number of samples for all factors; Right: a summary of the PVE explained by the components across all runs, where upper bound and the lower bound of the ribbon correspond to the maximum and minimum PVE, and the solid line correspond to the median. The components are inversely sorted by the median. (PDF) [file pcbi.1004791.s008.pdf]

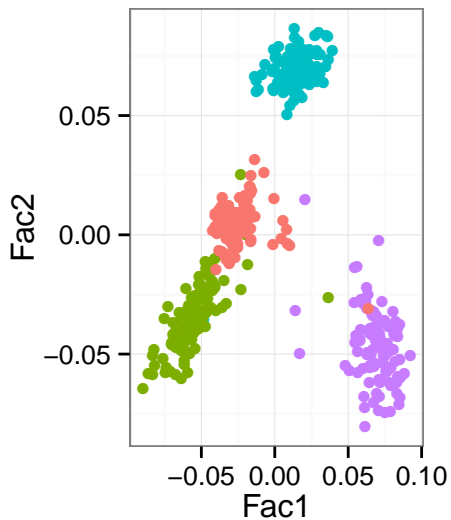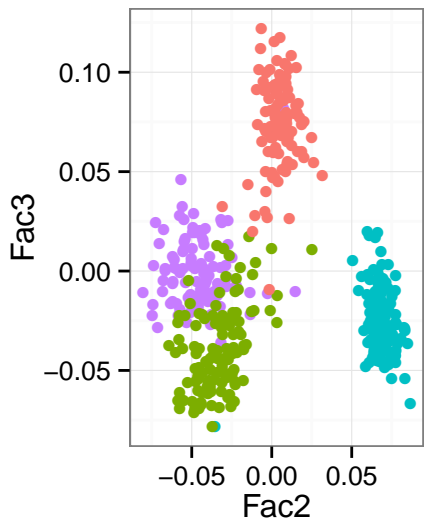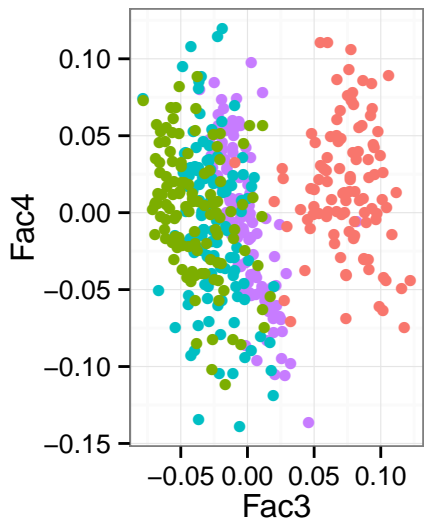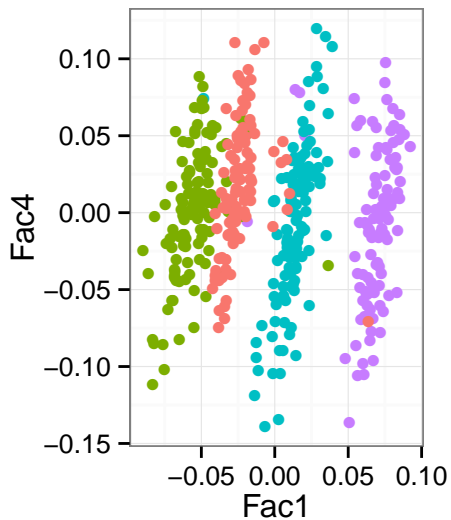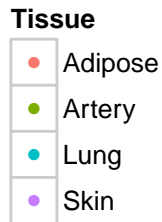

Supplement: S9 Fig — PC1 effectively separates the four tissue types. (PDF) [file pcbi.1004791.s009.pdf]

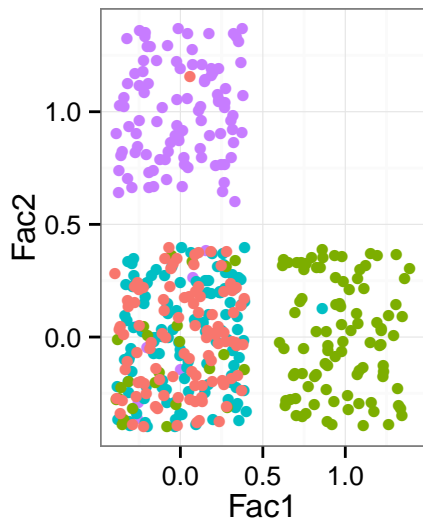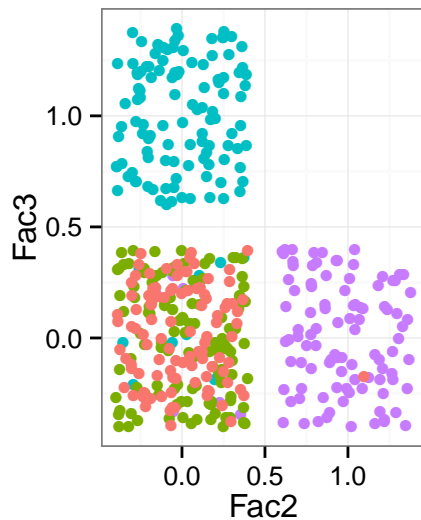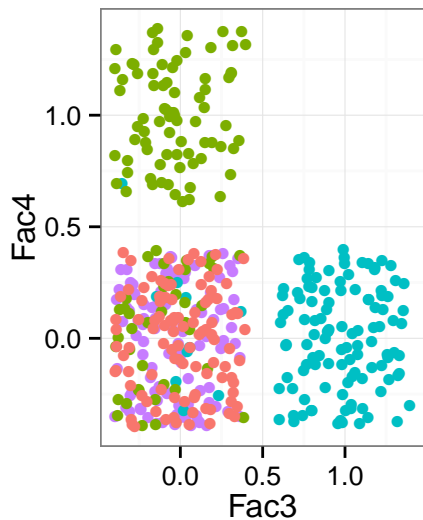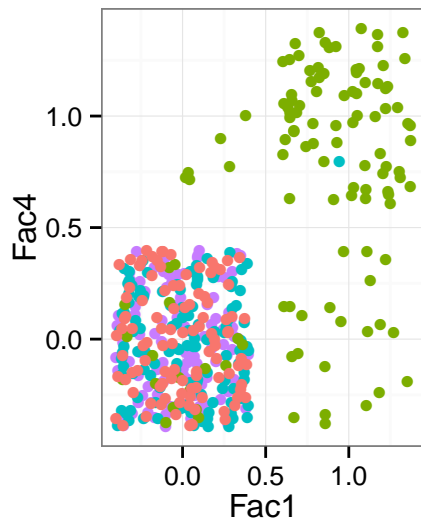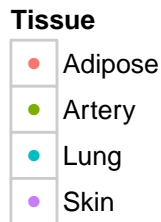

Supplement: S10 Fig — Each sample point is plotted with jitter to denote the density of each tissue in each included or excluded component. The four factors each capture variation in one (or a subset of one) of the tissues reasonably well, except for adipose. (PDF) [file pcbi.1004791.s010.pdf]

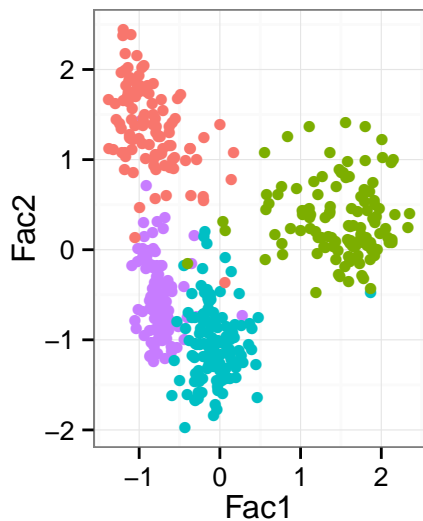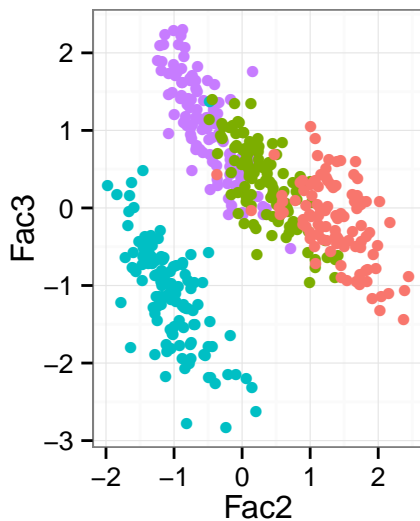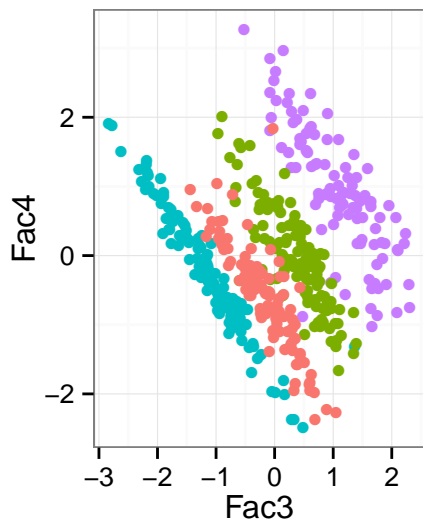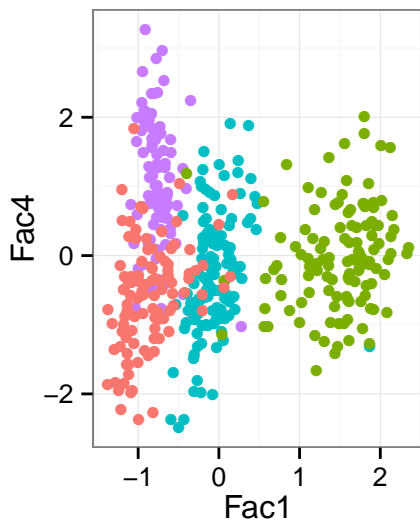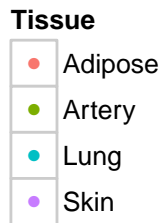

Supplement: S11 Fig — Across factors 1, 2, and 3, the four tissue types are effectively separated. (PDF) [file pcbi.1004791.s011.pdf]

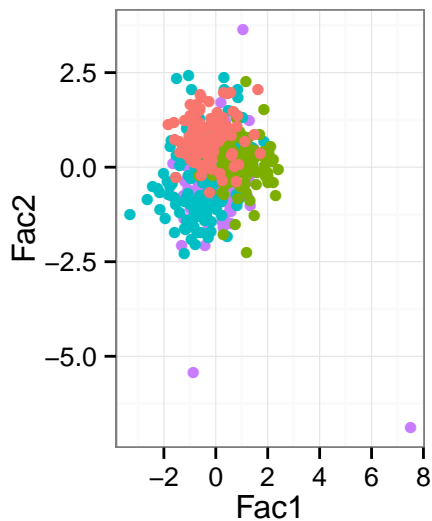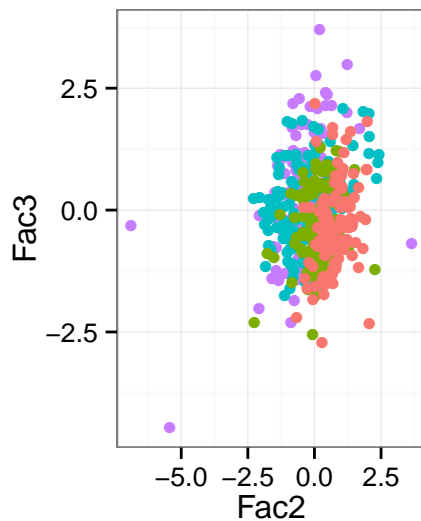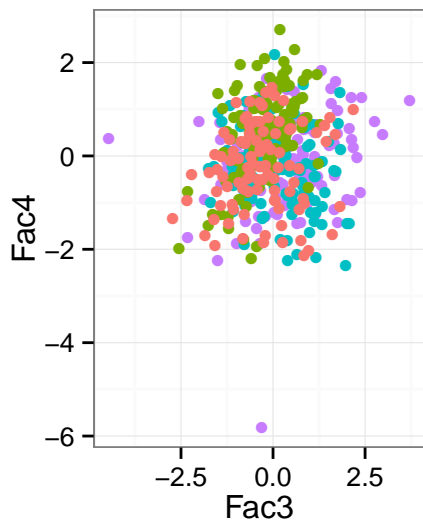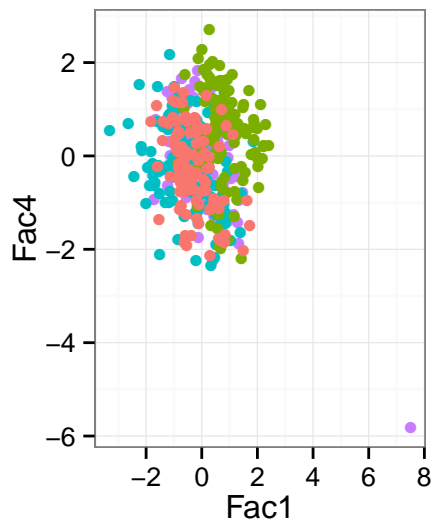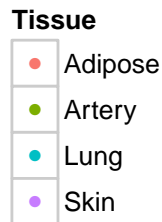

Supplement: S12 Fig — With 20 factors, Fabia is no longer able to separate the four tissue types because of limited sparsity. (PDF) [file pcbi.1004791.s012.pdf]

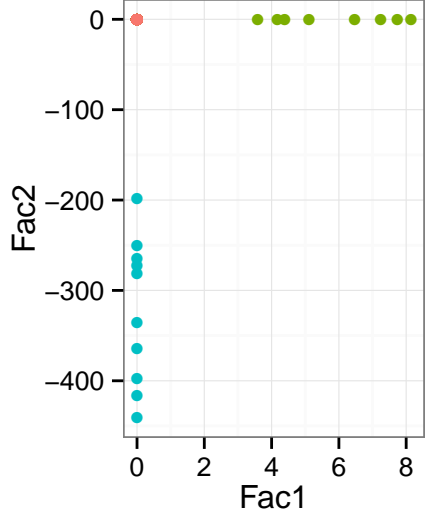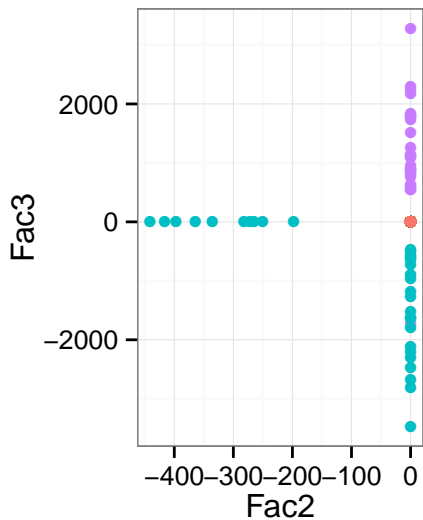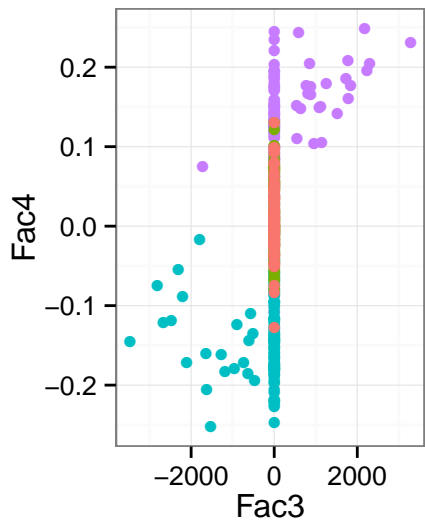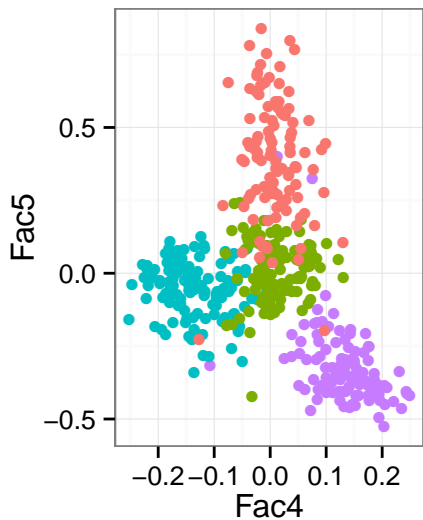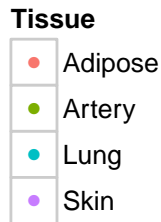

Supplement: S13 Fig — The substantial sparsity induced in BicMix is illustrated in these panels. Note that BicMix separates all four tissues in the first four factors. (PDF) [file pcbi.1004791.s013.pdf]
