# Supplementary material for: Context Specific and Differential Gene Co-expression Networks via Bayesian Biclustering
Source: PLoS Comput Biol. 2016 Jul 28;12(7):e1004791. doi: 10.1371/journal.pcbi.1004791 (PMC4965098; doi:10.1371/journal.pcbi.1004791)
Supplement: S2 Fig — The x- and y-axes represents the observed covariates; darker blue and red represent large magnitude correlations, whereas white represents no correlation. (PDF) [file pcbi.1004791.s002.pdf]

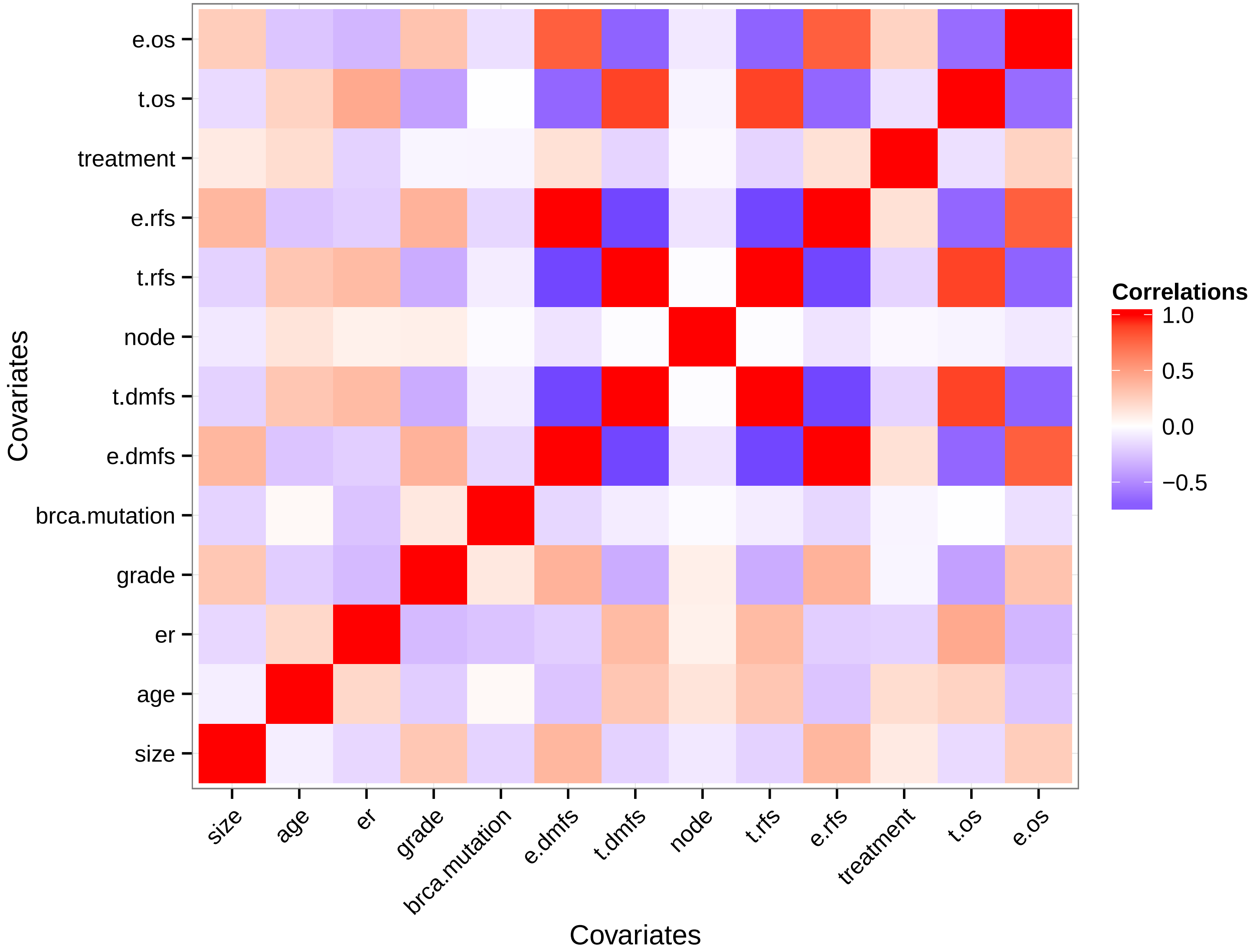

e.os
t.os
treatment
e.rfs
t.rfs
node
t.dmfs
e.dmfs
brca.mutation
grade
er
age
size
Covariates
size
age
er
grade
brca.mutation
e.dmfs
t.dmfs
node
t.rfs
e.rfs
treatment
t.os
e.os
Covariates
Correlations
1.0
0.5
0.0
−0.5
