# Supplementary material for: Context Specific and Differential Gene Co-expression Networks via Bayesian Biclustering
Source: PLoS Comput Biol. 2016 Jul 28;12(7):e1004791. doi: 10.1371/journal.pcbi.1004791 (PMC4965098; doi:10.1371/journal.pcbi.1004791)
Supplement: S7 Fig — Panel a: Gene co-expression network specific to smokers. Panel b: Gene co-expression network specific to non-smokers. (PDF) [file pcbi.1004791.s007.pdf]

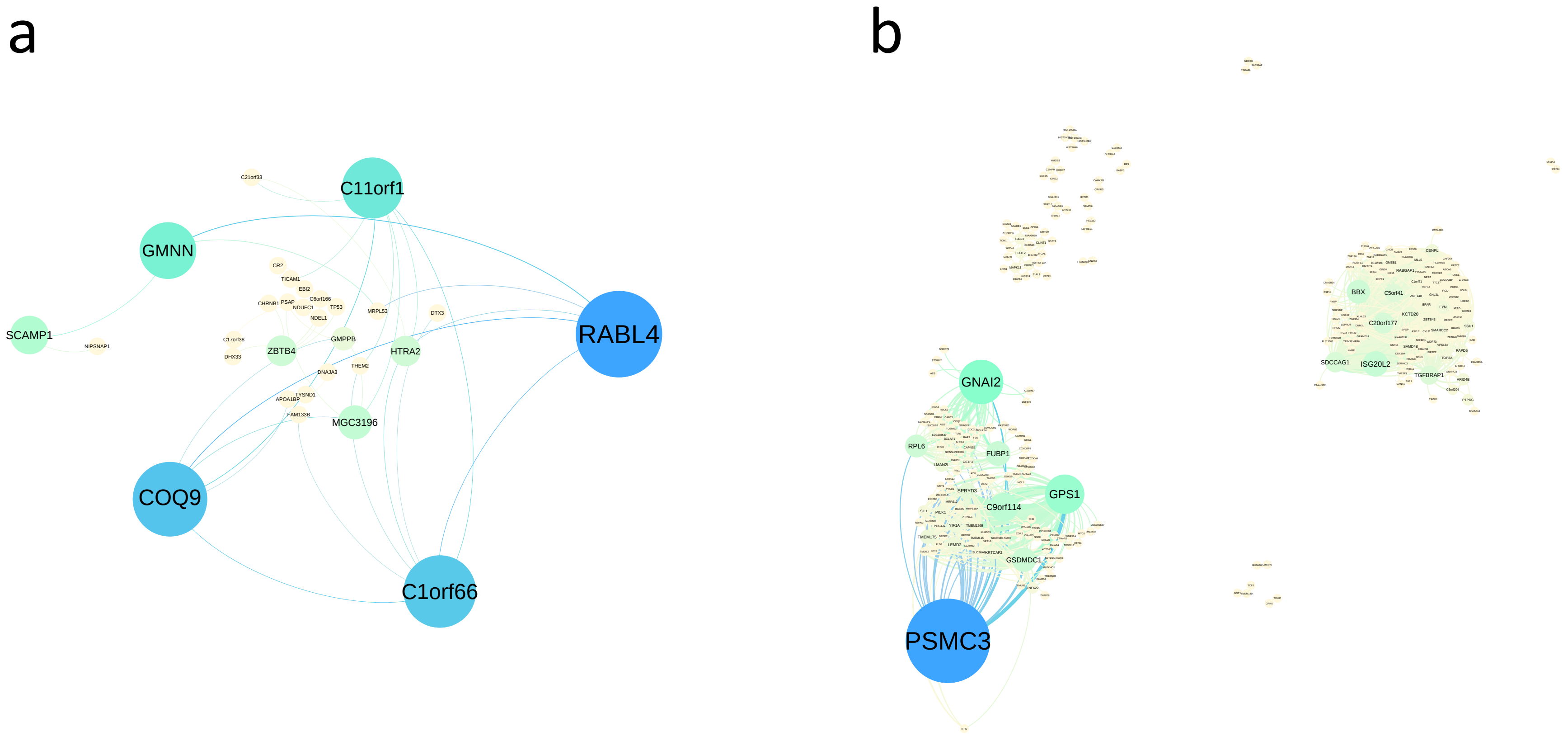

a
C21orf33
C11orf1
GMNN
SCAMP1
NIPSNAP1
CR2
TICAM1
EBI2
C6orf166
CHRNB1
PSAP
NDUFC1
TP53
NDEL1
MRPL53
DTX3
C17orf38
DHX33
ZBTB4
GMPPB
HTRA2
RABL4
DNAJA3
THEM2
TYSND1
APOA1BP
FAM133B
MGC3196
COQ9
C1orf66
b
GNAI2
RPL6
FUBP1
GPS1
SPRYD3
C9orf114
GSDMDC1
PSMC3
BBX
C5orf41
C20orf177
SDCCAG1
ISG20L2
TGFBRAP1
